# Impact of detecting potentially serious incidental findings during multi-modal imaging

**DOI:** 10.12688/wellcomeopenres.13181.3

**Published:** 2018-08-02

**Authors:** Lorna M Gibson, Thomas J Littlejohns, Ligia Adamska, Steve Garratt, Nicola Doherty, UK Biobank Imaging Working Group, Joanna M Wardlaw, Giles Maskell, Michael Parker, Roger Brownsword, Paul M Matthews, Rory Collins, Naomi E Allen, Jonathan Sellors, Cathie LM Sudlow

**Affiliations:** 1 Usher Institute of Population Health Sciences and Informatics, University of Edinburgh, Edinburgh, UK; 2 Clinical Trial Service Unit and Epidemiological Studies Unit, Nuffield Department of Population Health, University of Oxford, Oxford, UK; 3 UK Biobank Coordinating Centre, Stockport, UK; 4 Centre for Clinical Brain Sciences, University of Edinburgh, Edinburgh, UK; 5 Royal Cornwall Hospitals NHS Trust, Cornwall, UK; 6 Ethox Centre, Nuffield Department of Population Health, University of Oxford, Oxford, UK; 7 The Dickson Poon School of Law, King’s College London, London, UK; 8 Division of Brain Sciences, Department of Medicine, Hammersmith Hospital, Imperial College London, London, UK

**Keywords:** incidental findings, magnetic resonance imaging, dual-energy X-ray absorptiometry, false positives, false negatives, research ethics

## Abstract

**Background**: There are limited data on the impact of feedback of incidental findings (IFs) from research imaging.  We evaluated the impact of UK Biobank’s protocol for handling potentially serious IFs in a multi-modal imaging study of 100,000 participants (radiographer ‘flagging’ with radiologist confirmation of potentially serious IFs) compared with systematic radiologist review of all images.

**Methods**: Brain, cardiac and body magnetic resonance, and dual-energy x-ray absorptiometry scans from the first 1000 imaged UK Biobank participants were independently assessed for potentially serious IFs using both protocols. We surveyed participants with potentially serious IFs and their GPs up to six months after imaging to determine subsequent clinical assessments, final diagnoses, emotional, financial and work or activity impacts.

**Results**: Compared to systematic radiologist review, radiographer flagging resulted in substantially fewer participants with potentially serious IFs (179/1000 [17.9%] versus 18/1000 [1.8%]) and a higher proportion with serious final diagnoses (21/179 [11.7%] versus 5/18 [27.8%]). Radiographer flagging missed 16/21 serious final diagnoses (i.e., false negatives), while systematic radiologist review generated large numbers of non-serious final diagnoses (158/179) (i.e., false positives). Almost all (90%) participants had further clinical assessment (including invasive procedures in similar numbers with serious and non-serious final diagnoses [11 and 12 respectively]), with additional impact on emotional wellbeing (16.9%), finances (8.9%), and work or activities (5.6%).

**Conclusions**: Compared with systematic radiologist review, radiographer flagging missed some serious diagnoses, but avoided adverse impacts for many participants with non-serious diagnoses. While systematic radiologist review may benefit some participants, UK Biobank’s responsibility to avoid both unnecessary harm to larger numbers of participants and burdening of publicly-funded health services suggests that radiographer flagging is a justifiable approach in the UK Biobank imaging study. The potential scale of non-serious final diagnoses raises questions relating to handling IFs in other settings, such as commercial and public health screening.

## Introduction

UK Biobank ( www.ukbiobank.ac.uk) is a major resource for research into the determinants of a wide range of serious and life-threatening diseases, to improve their prevention, diagnosis and treatment ^1^. It is a prospective study which recruited 500,000 men and women aged 40–69 across the UK between 2006 and 2010 ^1^. It includes extensive questionnaire and physical measurement data from the baseline visit, biological samples (with genotyping and biomarker assay data), longitudinal follow-up data from national health-related datasets and additional information from remote monitoring and web-based questionnaires.

The UK Biobank imaging study aims to perform brain, cardiac and body magnetic resonance imaging (MRI), dual-energy x-ray absorptiometry (DXA) and carotid Doppler ultrasound in 100,000 UK Biobank participants in dedicated imaging centres over seven years ( http://imaging.ukbiobank.ac.uk/). By November 2017, over 20,000 participants had attended an imaging assessment visit ( http://imaging.ukbiobank.ac.uk/), making it already the world’s largest ever multi-modal imaging study ^2^.

Incidental findings (IFs), defined as ‘findings discovered in the course of research that are beyond the aims of the study,’ ^3^ are a predictable consequence of much research, and studies need appropriate protocols for handling them ( https://wellcome.ac.uk/funding/managing-grant/wellcome-trust-policy-position-health-related-findings-research/) ^4^. IFs are particularly pertinent to the UK Biobank imaging study given its large scale and the potential seriousness of IFs that may be detected. While clinical care and screening programmes aim to provide clinical benefit to patients, research studies have the primary aim of producing generalisable knowledge. Nevertheless, while research studies do not aim to benefit participants directly, they are obliged to minimise potential harms to participants and the wider public. Hence, although the UK Biobank imaging study aims to collect research data, rather than to detect or diagnose serious disease, it does require a protocol to handle IFs should they arise.

The UK Biobank imaging IFs protocol was developed as a pragmatic, scalable process, aiming to produce the best possible resource for biomedical research while minimising any potential harms for 100,000 largely asymptomatic UK Biobank participants. UK Biobank reviewed current practice, the extensive literature ^3, 5, 6^ and relevant published guidance ( https://www.rcr.ac.uk/publication/management-incidental-findings-detected-during-research-imaging), sought independent legal advice, and consulted with its independent Ethics and Governance Council, the UK’s Royal College of Radiologists and Society and College of Radiographers, funders, relevant experts and leading imaging research projects (including the Multi-Ethnic Study of Atherosclerosis [ http://www.hopkinsmedicine.org/heart_vascular_institute/clinical_trials/preventive/mesa.html], the Reykjavik Heart Study [ http://www.hjartarannsokn.is/index.aspx?GroupId=406], the Rotterdam Scan Study ^7^ and the German National Cohort [ http://nako.de/]) ^2^. Key contextual factors considered were the non-clinical setting of the imaging visit, in which the scanning sequences are optimised for research use rather than clinical diagnosis, and the nature of the participants’ existing consent (in particular the approach to the feedback of IFs). However, cost effectiveness was not considered relevant ^2^.

The UK Biobank imaging IFs protocol involves feedback to participants and their general practitioners (GPs) when a radiographer observes a potentially serious IF during image acquisition that is subsequently confirmed by a specialist radiologist. UK Biobank defines a potentially serious IF for these purposes as one indicating the possibility of a condition which, if confirmed, would carry a real prospect of seriously threatening life span, or of having a substantial impact on major body functions or quality of life.

### The need for evidence to inform IFs policy

Limited data exist on the impact of feedback of IFs on participants and health services ^8– 11^, and on how these vary by different policies for handling IFs. Most published data on opinions of receiving such feedback are based on hypothetical scenarios, rather than studies of research participants who have actually received feedback ^12– 14^. It is often assumed that early observation on imaging of presumed disease (prior to clinical presentation) is inevitably beneficial, but data on final clinical diagnosis and the impact of feedback of IFs are scarce ^15^. Such data would inform debates about these assumptions, and the design of appropriate, acceptable protocols to handle IFs detected in research, public health screening or commercial imaging settings.

In this evaluation of the first 1000 participants in the UK Biobank imaging study, we assessed the number and types of potentially serious IFs detected and their final clinical diagnoses, comparing the UK Biobank imaging IFs protocol with systematic radiologist review of all of the images. We also assessed the impact of providing feedback about potentially serious IFs on participants, their friends, families and health services, with respect to: clinical assessments undertaken; emotional wellbeing, finances, work and daily activities; and participants’ and their general practitioners’(GP) opinions about receiving feedback.

## Methods

### Participants

Existing participants of the UK Biobank cohort study who lived within about 100 miles of UK Biobank’s first imaging centre in Stockport were invited to participate in the UK Biobank imaging study. The invitation contained a link to the UK Biobank imaging study website ( http://imaging.ukbiobank.ac.uk/), and willing participants were asked to telephone the Participant Recruitment Centre where they could ask questions about the study and answer pre-screening safety questions. Participants were excluded if they had metal inside their body or an implanted medical device which could create imaging artefacts or pose a risk during MRI, if they were likely to find it difficult to lie still, or if they were unlikely to tolerate the imaging due to known claustrophobia.

### Consent

All participants received written information about the imaging study, including details about the UK Biobank imaging IFs protocol, and provided consent before taking part, including consent for UK Biobank to inform them and their GP if a potentially serious IF was identified ( Supplementary File 1). We surveyed all participants with a questionnaire two days after their imaging assessment to assess their understanding of the information and consent process ( http://www.ukbiobank.ac.uk/resources/).

### Imaging

Participants underwent a 30 minute brain MRI (3.0 Tesla Skyra scanner, Siemens, Erlangen, Germany), a 30 minute non-contrast cardiac and body MRI (1.5 Tesla Aera scanner, Siemens, Erlangen, Germany) from neck to knees ( Supplementary File 2), and a 15 minute DXA scan (iDXA, General Electric, New York, United States of America) of whole body, lumbar spine and hip, with lateral vertebral fracture assessment. Participants also underwent carotid doppler ultrasound, but this was not considered to have the potential to yield potentially serious IFs ( Supplementary File 3). Imaging protocols were optimised for research purposes and did not constitute standard diagnostic examinations.

### List of potentially serious IFs

UK Biobank consulted radiologists, reviewed the literature, and considered the German National Cohort’s list of imaging IFs ^16^ to develop a list of IFs considered to be potentially serious, as well as examples of those not considered serious ( Supplementary File 3). Both radiographers and reporting radiologists used this list in conjunction with UK Biobank’s definition of a potentially serious IF when judging whether any observed IF was potentially serious or not.

### Two protocols for handling IFs

Images from the first 1000 participants were assessed using two protocols which ran simultaneously. Under the UK Biobank IFs protocol (‘radiographer flagging’), if a radiographer noticed a potentially serious IF during image acquisition and quality assessment, the relevant set of images was flagged for subsequent review by a radiologist. Under ‘systematic radiologist review’, all images were systematically reviewed by a radiologist. Radiographers were trained in the relevant imaging protocols but did not receive specific training in image interpretation as UK Biobank is a research resource and conducts research imaging. The radiographers were not instructed to actively look for, or to avoid looking for IFs; rather, they were instructed that should they happen to notice a concerning finding, they should flag it for review. As such, UK Biobank does not aim to provide any form of health service, including image interpretation. Radiologists and radiographers were aware of the comparison study, but were blind to each other’s opinions. To aid interpretation of images assessed either during systematic radiologist review, or those flagged by radiographers, we provided reporting radiologists with data collected during the imaging visit on the participant’s age, sex, body mass index, self-reported smoking status, alcohol consumption, medical history and medications.

Within a few weeks of their imaging visit, we wrote to all participants who had a potentially serious IF reported by a radiologist, whether it had been both flagged by a radiographer and confirmed by a radiologist (radiographer flagging) or detected by a radiologist during systematic review of all images (systematic radiologist review). We explained that a potentially serious abnormality (or, sometimes, abnormalities) had been observed, and advised the participant to visit his/her GP for advice about any further action required ( Supplementary File 4). We also wrote to these participants’ GPs, providing a copy of the radiologist’s report and, if requested, copies of the relevant scans ( Supplementary File 5).

### Questionnaires to participants with potentially serious IFs and their GPs

We surveyed participants with potentially serious IFs approximately six weeks after writing to them and their GP and approximately six months after their imaging visit to assess the impact of this information. Both participant questionnaires collected data on clinical assessment (blood tests, imaging, specialty referral, changes in medication, invasive procedures and operations), final diagnoses, and opinions on receiving feedback and participating in the imaging study, with additional questions at six months on emotional wellbeing, insurance, finances, work and activities. We also surveyed GPs at six months about clinical assessments, final diagnoses (including copies of any relevant clinical correspondence) and their perceptions of the impact on their patients of receiving feedback ( http://www.ukbiobank.ac.uk/resources/). We reconciled multiple responses on similar items from the three questionnaires by prioritising ‘yes’ responses and included data from coding of free text responses ( http://www.ukbiobank.ac.uk/resources/).

### Determining final clinical diagnoses

Because there is a paucity of empirical evidence on the natural history and final diagnoses of IFs, ^15^ and no validated risk scores for quantitatively determining the risk to lifespan of particular IFs which are detected on research imaging, our classification of final diagnoses as ‘serious’ was based on clinical judgement. A consultant physician and an experienced speciality clinical radiology trainee independently classified final diagnoses for each participant who received feedback about a potentially serious IF, by reviewing all available questionnaire data together with additional relevant clinical information from further correspondence or telephone calls with the participant and/or their GP. Working from the definition of a potentially serious IF, we classified final clinical diagnoses as: serious if they were likely to significantly threaten lifespan or have a major impact on quality of life or major body functions; not serious if this was not the case, or if the available data suggested that the diagnosis was already known; and uncertain if there were insufficient data to classify as serious or not. We classified participants with more than one potentially serious IF according to their most serious final diagnosis. Given this inherent subjectivity in the classification of serious final diagnoses, we measured the repeatability of the clinical judgements of final diagnoses severity by calculating the percentage of participants in whom both classifying doctors agreed on their initial classification. We resolved disagreements through discussion and mutual consensus.

### Qualitative study

To provide additional context, UK Biobank commissioned a social research company (TNS-BMRB; www.tns-bmrb.co.uk) to conduct a parallel qualitative study. This aimed: (1) to explore participants’ understanding of and opinions about the process of consent relating to feedback of potentially serious IFs through deliberative group discussions with two groups of around 10 participants each (a more and a less affluent group) prior to their imaging assessment; and (2) to assess views on the process and impact of receiving feedback through one-to-one interviews with 15–20 participants (including more and less affluent male and female participants) with IFs on different imaging modalities, and with both serious and non-serious final clinical diagnoses. Further details of the methods of recruitment, interview content and qualitative analysis methods are available at http://www.ukbiobank.ac.uk/resources/.

### Statistical analyses

We summarised data from questionnaires as counts and proportions. We compared groups using chi-squared or Fisher’s exact tests for proportions and Student’s independent t-test for continuous variables. We considered p values of <0.05 to be statistically significant and analysed data using Microsoft Excel 2013 and SPSS Statistics version 21.

### Ethics approval

UK Biobank obtained approval specifically for the imaging study, participant information and consent materials and this evaluation, including surveying participants and their GPs (North West Research Ethics Committee, Reference Number: 11/NW/0382).

## Results

**Figure 1.**
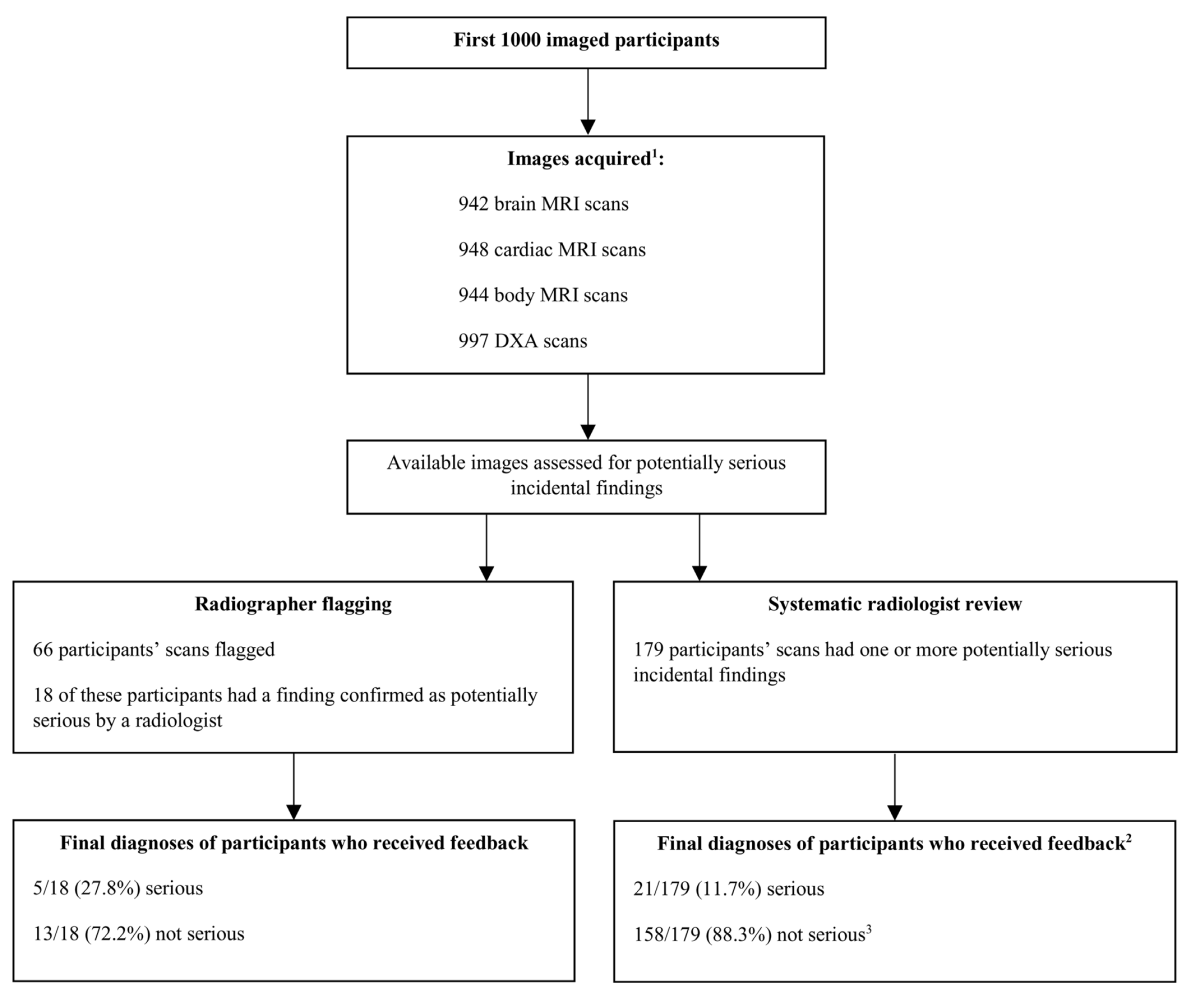
Participant flowchart. MRI = magnetic resonance imaging, DXA = dual energy x-ray absorptiometry. ^1^68 participants had incomplete imaging: 18 underwent DXA but not MRI due to safety issues, 50 did not complete all MRI (28 due to claustrophobia, 13 due to scanner failure, nine for other reasons). ^2^Final diagnosis assigned to participants with more than one potentially serious incidental finding was the most serious (serious>uncertain>not serious). ^3^Three of these participants had uncertain final diagnoses, see Supplementary File 7.

The first 1000 eligible participants were imaged between April and October 2014. Their mean age was 62 (range 44–77) years, and 524 (52.4%) were female. Each MRI imaging modality was conducted in >94% participants, and DXA in >99% ( Figure 1).

### Understanding of consent

Around 60% of the first 1000 participants (607/1000) completed the questionnaire assessing understanding of consent. The vast majority correctly understood that they would not receive their scans or results at the end of the imaging visit (540/607, 86.7%) and that they would not be told about any potentially serious IF during the visit (89.0%), but around a quarter incorrectly thought that they could choose whether or not to be informed about any potentially serious IF (158/607, 26.0%) ( Supplementary File 6).

### Potentially serious IFs

Radiographers flagged 66 potentially serious IFs in 66 (6.6%) participants. Of these, 18 (1.8%) were confirmed as potentially serious by radiologists. Radiologists detected potentially serious IFs in 179 (17.9%) participants ( Figure 1), who included the 18 participants with potentially serious IFs flagged by radiographers. Participants with potentially serious IFs were slightly older than those without (mean age 63 versus 61 years, p=0.03), but their sex distribution did not differ significantly (55.3% vs 51.8% female, p=0.4).

### Final diagnoses

Data on final diagnoses were available from one or more questionnaires, clinical correspondence and/or telephone contact in 176/179 (98.3%) participants. The two doctors agreed on the per-participant classification of final diagnoses in 172/179 (96.1%) cases. The seven cases of initial disagreement were readily resolved by discussion.

A higher proportion of participants with potentially serious IFs had serious final diagnoses (i.e. true positives) with radiographer flagging (5/18, 27.8%) than with systematic radiologist review (21/179, 11.7%, Figure 1, Table 1). A higher proportion and substantially greater absolute number had non-serious final diagnoses (i.e. false positives) with systematic radiologist review (158/179, 88.3%) than radiographer flagging (13/18, 72.2%). However, radiographer flagging missed 16 of the 21 participants with a serious final diagnosis detected by systematic radiologist review (i.e. false negatives) ( Figure 1, Table 1, Supplementary File 7).

**Table 1.** Clinical seriousness of final diagnoses of 179 participants by detection method and imaging modality.

Method of detection and imaging modality	Clinical seriousness of final diagnosis (n participants)			% of 1000 imaged participants with ≥ 1 PSIF detected	% of participants in whom a PSIF predicted a serious final diagnosis
	Serious	Non-serious ^1^	Total		
**Radiographer flagging**					
Brain MRI	2	2	4	0.4	50.0
Cardiac MRI	3	2	5	0.5	60.0
Body MRI	0	8	8	0.8	0.0
DXA	0	1	1	0.1	0.0
> 1 modality	0	0	0	0.0	0.0
**Total (any modality)**	5	13	18	1.8	27.8
**Systematic radiologist** **review**					
Brain MRI	4	14	18	1.8	22.2
Cardiac MRI	13	54	67	6.7	19.4
Body MRI	3	65	68	6.8	4.4
DXA	1	10	11	1.1	9.1
> 1 modality ^2^	0	15	15	1.5	0.0
**Total (any modality)**	21	158	179	17.9	11.7

PSIF = potentially serious incidental finding, MRI = magnetic resonance imaging, DXA = dual energy X-ray absorptiometry ^1^ Includes three participants whose final diagnoses remained uncertain as of April 2016: one participant with a lung nodule was still under assessment; another participant with a lung nodule had been diagnosed with lymphoma, but it remained unclear whether the nodule was related to the lymphoma or not; and we were unable to contact one participant to determine the final diagnosis of DXA appearances suggesting a crush fracture. ^2^ Fifteen participants had more than one non-serious final diagnosis arising from more than one modality.

The numbers and proportions of participants with potentially serious IFs and with serious versus non-serious final diagnoses varied substantially by imaging modality. Most of the 158 false positives generated by systematic radiologist review were identified on cardiac or body MRI (54 on cardiac and 65 on body [mainly abdominal] MRI; Table 1). Participants with potentially serious IFs from brain and cardiac MRI were more likely to have a serious final diagnosis (around half under radiographer flagging, and 20% under systematic radiologist review) than those with potentially serious IFs from the other imaging modalities ( Table 1).

Systematic radiologist review generated 217 potentially serious IFs in 179 participants. More than one potentially serious IF occurred in 33 participants (28 had two and five had three), although no participant had more than one serious final diagnosis. The 21 serious final diagnoses included aortic aneurysms, tumours, structural and functional cardiac disease, and osteoporotic fractures, while non-serious final diagnoses comprised benign lesions, diagnoses already known to the participant and/or their GP, and suspected lesions which were not confirmed. Radiographer flagging detected five of these 21 serious final diagnoses (one arachnoid cyst with hydrocephalus, one meningioma compressing brainstem, and three thoracic aortic aneurysms), and missed 16/21 (two pituitary tumours, two thoracic aortic aneurysms, three lung tumours, two cardiomyopathies, and one each of: atrial fibrillation, coronary heart disease, heart block with left ventricular impairment, abdominal aortic aneurysm, gastrointestinal stromal tumour, pancreatic neuroendocrine tumour, and an osteoporotic crush fracture) (See Supplementary File 7).

### Follow-up questionnaires

Each of the three follow-up questionnaires was returned for ≥70% of 179 participants with a potentially serious IF; at least one questionnaire was returned for 93.3% and all three for 45.8% ( Table 2). Denominators varied for different types of clinical assessment and impact due to different proportions of completed responses to the relevant questions ( Table 3).

**Table 2.** Available questionnaires returned by 179 participants and their GPs.

	n participants (%)
Six-week participant questionnaire	132 (74)
Six-month participant questionnaire	125 (70)
Six-month GP questionnaire	125 (70)
At least one questionnaire returned ^1^	167 (93)
All three questionnaires returned	82 (46)

^1^At least one of a six-week participant, six-month participant, or six-month GP questionnaire

**Table 3.** Clinical assessment, impact on participants, and opinions relating to feedback of potentially serious incidental findings ^1^.

	Clinical seriousness of final diagnosis ^2^						p value (Fisher’s exact test) for difference between those with a serious versus non-serious final diagnosis
	Serious		Non-serious ^3^		Total		
Impact or opinion	n/N ^4^	%	n/N ^4^	%	n/N ^4^	%	
**Clinical assessment as reported by** **participant, GP or both**							
Contact between participants and their GP by six months following feedback	20/20	100	146/146	100	166/166	100	**-**
Blood tests	6/20	30.0	44/150	29.3	50/170	29.4	1.0
Imaging	17/20	85.0	117/150	78.0	134/170	78.8	0.6
Other tests	3/20	15.0	12/150	8.0	15/170	8.8	0.4
Referral	17/20	85.0	92/150	61.3	109/170	64.1	0.05
Change of medication	8/18	44.4	9/144	6.3	17/162	10.5	<0.0001
Invasive procedure or operation	11/18	61.1	12/144	8.3	23/162	14.2	<0.0001
Any of the above clinical assessment	20/20	100	133/150	88.7	153/170	90.0	0.2
**Participants’ opinions**							
Any impact on:							
Emotional wellbeing ^5^	7/15	46.7	14/109	12.8	21/124	16.9	0.004
Insurance or finances ^6^	5/15	33.3	6/109	5.5	11/124	8.9	0.004
Work or activities of daily living ^7^	4/15	26.7	3/109	2.8	7/124	5.6	0.004
How their health compared to before the imaging visit							
Much better or a little better	0/15	0.0	9/109	8.3	9/124	7.3	
The same	9/15	60.0	95/109	87.2	104/124	83.9	0.0007
Much worse or a little worse	6/15	40.0	5/109	4.6	11/124	8.9	
I am glad that:							
UK Biobank told me about a potentially serious incidental finding ^8^	16/16	100.0	126/129	97.7	142/145	97.9	1.0000
I took part in the UK Biobank imaging study ^9^	17/17	100.0	130/131	99.2	147/148	99.3	1.0000
**GPs’ opinions**							
Impact on the participant’s emotional wellbeing:							
Positive or very positive	6/11	54.5	10/88	11.4	16/99	16.2	
No impact	1/11	9.1	44/88	50.0	45/99	45.5	0.001
Negative or very negative	4/11	36.4	34/88	38.6	38/99	38.4	
The net impact to the participant							
Net benefit	10/11	90.9	41/75	54.7	51/86	59.3	0.02
Net harm	1/11	9.1	34/75	45.3	35/86	40.7	
**Participant and GP opinions**							
“Participants should always be told about potentially serious incidental findings”							
Participants who agreed with this statement ^10^	6/17	35.3	49/132	37.1	55/149	36.9	1.0
GPs who agreed with this statement	6/9	66.7	55/85	64.7	61/94	64.9	1.0

-Test not performed ^1^ Based on combined responses to relevant questions from the six-week and six-month participant questionnaires, and the six-month GP questionnaire ^2^ 217 potentially serious incidental findings (IFs) were detected in 179 participants. For participants with more than one potentially serious IF, clinical severity of the final diagnosis per participant indicates the most severe diagnosis (serious > uncertain > not serious) of their potentially serious IFs ^3^ Including three participants for whom the final diagnosis remained uncertain by April 2016 (see Supplementary File 7 for details) ^4^ Denominators vary due to differences in questionnaire return rates and whether or not the relevant questions had been answered on returned questionnaires. ^5^ Any impact on either the emotional wellbeing of the participant, their friends, or their family, or on family life (combined responses across several related questions) ^6^ Any impact on either the cost or ability of obtaining travel, or health or life insurance or on their overall financial situation (combined responses across several related questions) ^7^ Any impact on having to take time off work, change job or retire, or have help for activities of daily living (combined responses across several related questions) ^8^ This question was asked on both the six-week and the six-month participant questionnaires. 145 participants answered the question at least once and formed the denominator. 98 of 100 who answered the question both times did so consistently (they were glad to have been told on both occasions) and were included in the numerator. Two of these 100 participants (both with final non-serious diagnoses) gave different answers on each questionnaire (one was glad to have been told at six weeks, but by six months would rather not have been told, while the other would rather not have been told at six weeks but was glad to have been told at six months). One further participant (who returned a single six-month participant questionnaire) reported that they would rather not have been told about their potentially serious IF, which was finally diagnosed as a non-serious condition. ^9^ This question was asked on both the six-week and the six-month participant questionnaires. 148 participants answered the question at least once and formed the denominator. Answers from the 101 participants who returned both questionnaires and answered the question both times were all consistent ^10^ This question was asked on both the six-week and the six-month participant questionnaires. 149 participants answered the question at least once and formed the denominator. 69 of the 105 participants who answered the question both times did so consistently and were included in the numerator. 36 gave different answers on each questionnaire: 22 changed their view from ‘should always be told’ to 21 ‘should be able to choose’ and one ‘no opinion’; 14 changed from ‘should be able to choose,’ to 11 ‘should always be told’ and three ‘other option’.

### Clinical assessment

All participants with follow-up questionnaire data had contacted their GP. Almost all had some form of clinical assessment (153/170 [90.0%]), most frequently blood tests (29.4%), further imaging (78.8%) or specialist referral (64.1%), with smaller proportions having other tests (8.8%), change of medication (10.5%) or an invasive procedure or operation (14.2%) ( Table 3). The proportions having each type of clinical assessment were generally higher for those with a serious compared with non-serious final diagnosis, particularly medication changes (44.4% serious versus 6.3% non-serious) and invasive procedures (61.1% versus 8.3%). However, the absolute numbers having clinical assessment were far higher among the many more participants with non-serious final diagnoses. Of the 153 participants reporting some form of clinical assessment, 133 had a non-serious final diagnosis, suggesting that further clinical assessment might not have been necessary ( Table 3).

Of particular note, similar absolute numbers of participants had invasive, potentially harmful, procedures irrespective of whether their final diagnosis was considered to be serious (n=11) or non-serious (n=12) ( Supplementary File 8). The clinical management of the participants with a serious final diagnosis is summarised in Supplementary File 9.

### Impact on participants

Feedback about a potentially serious IF also had an impact (presumed to be adverse) on participants’ emotional wellbeing (21/124, 16.9%), insurance or finances (11/124, 8.9%), and work or activities of daily living (7/124, 5.6%). The proportion of participants reporting an impact on emotional wellbeing was higher among those with a serious final diagnosis, but the absolute numbers were higher among those with a non-serious final diagnosis, for whom these impacts could be considered to constitute net harm ( Table 3). In addition to the 21 reporting an impact on emotional wellbeing in response to the relevant survey question, participants and/or their GPs spontaneously mentioned worry within questionnaire free-text responses for a further 62 participants (examples shown in Box 1).

Box 1. **QUOTATIONS FROM PARTICIPANTS AND THEIR GENERAL PRACTITIONERS (GP)**

Participant with a non-serious final diagnosis, six-week questionnaire: “Better to know, but I did feel anxious for a few weeks.”

Participant with a serious final diagnosis, six-month questionnaire: “Life has been a physical & emotional roller-coaster since then, both for myself, family & friends. A serious risk of death on the operating table, and considering the consequences for my wife. All-in-all, I feel as if I was mugged by medical technology.”

GP of a participant with a non-serious final diagnosis: “[The patient] was asymptomatic. In normal practice no investigation would be performed - this has led to unnecessary anxiety and tests.”

GP of a participant with a non-serious final diagnosis: “Concerns over use of health resources regarding this. Using GP and secondary care time with potential [upper gastrointestinal endoscopy] +/- associated risks of this procedure. This for symptoms that the patient is not too concerned with at present.”

Most participants receiving feedback reported no change in their health since the imaging visit (104/124, 83.9%). Similar absolute numbers among those with serious versus non-serious final diagnoses had worse health (6/15, 40.0% versus 5/109, 4.6%), while a few of those with a non-serious final diagnosis (but none with a serious final diagnosis) reported better health (9/109, 8.3%, Table 3).

### Opinions on receiving feedback

Almost all participants reported being glad to be told about their potentially serious IF (142/145 (97.7%) ( Table 3). Nonetheless, GPs who responded reported that a higher proportion of participants had experienced negative versus positive impact on emotional wellbeing (38/99, 38.4% versus 16/99, 16.2%), with most of the negative impact occurring among those with non-serious final diagnoses ( Table 3). GPs also spontaneously highlighted concerns about use of health resources to manage asymptomatic people within their free-text questionnaire responses ( Box 1). However, the responding GPs believed that a slightly higher proportion of participants had experienced net benefit compared to net harm (51/86, 59.3% versus 35/86, 40.7%).

A higher proportion of responding GPs (61/94, 64.9%) than participants (55/149, 36.9%) thought participants should be always told about a potentially serious IF ( Table 3). Since participants were asked both at six weeks and at six months about this, we were able to assess whether the answers of 105 participants who responded on both occasions changed over time. While 69 had consistent responses, 36 changed their views (n=21, Table 3: footnote 10).

### Results of the qualitative study

Deliberative group discussions about consent involved a group of 10 ‘more affluent’ participants (Townsend score <-2, four female, mean age 61, SD 9.1 years), and a group of 11 ‘less affluent’ participants (Townsend score >0, six female, mean age 66 years). One-to-one interviews involved an additional 21 participants who received feedback about a potentially serious IF (13 ‘more affluent’, 13 female, mean age 66 years). Analysis of the interview data revealed that participants were motivated to attend the imaging study by altruism, to experience MRI scanning first-hand (in case they needed to attend for investigations for a medical concern later in life), and to receive feedback about potentially serious IFs. Participants could not always recall precise details of the consent process with respect to feedback of IFs, but they were generally unconcerned about this as they trusted UK Biobank to act appropriately. One-to-one interviews further demonstrated that the implications of receiving feedback were not fully understood until after the event, that feedback resulted in short-term anxiety, and that participants tended to assume the worst on receiving feedback; indeed, some were surprised that the final diagnosis might be non-serious, having anticipated a diagnosis of cancer, an aneurysm or a serious heart condition. Further details of the qualitative study results are available at http://www.ukbiobank.ac.uk/resources/.

## Discussion

Compared to systematic review of images by radiologists, the UK Biobank IFs protocol (radiographer flagging) resulted in approximately 10-fold fewer participants with non-serious diagnoses (i.e., false positives), but missed 16/21 potentially serious IFs that were diagnosed ultimately as a serious disease (i.e. false negatives).

**Figure 2.**
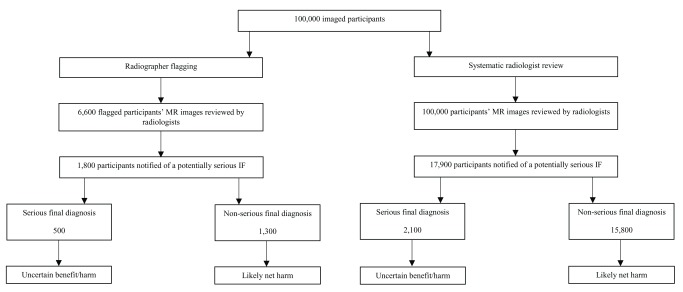
Extrapolation of this study’s findings to the 100,000 UK Biobank imaging study participants. MR = magnetic resonance, IF = incidental finding.

Extrapolation of our results to the 100,000 participants who will be imaged by UK Biobank over the next few years suggests that systematic radiologist review would generate 15,800 false positives, compared with 1,300 under the UK Biobank IF protocol (radiographer flagging), and would detect serious diagnoses in 2,100 participants compared with 500 under radiographer flagging ( Figure 2).

Systematic radiologist review in our study generated a prevalence of potentially serious IFs of 17.9%. The prevalence in other whole-body MRI studies of healthy populations ranged from 12.8% to 57.6% ^17– 20^. Since those studies used similar MRI sequences applied to similar tissue volumes, variations in prevalence are most likely to have arisen from differences in the definition of IFs, or in the age and other characteristics of the imaged populations.

Almost all participants with potentially serious IFs had subsequent clinical assessment, resulting in large numbers of investigations, referrals and procedures. Many of these were, with hindsight, unnecessary, with risk of direct harm as well as cost implications. Impact on emotional wellbeing, insurance or finances, and on work or daily activities were reported by a higher proportion of participants with serious final diagnoses, but affected a higher absolute number of participants without serious final diagnoses. In keeping with these results, over half of participants in the Study of Health in Pomerania who received feedback of an IF detected on whole-body MRI reported psychological distress ^8^.

Only around one-third of our participants believed that participants should always be told about potentially serious IFs. Similar proportions of participants with serious and participants with non-serious final diagnoses expressed this opinion. However, almost a quarter of participants changed their opinion over the few months between the six-week and six-month questionnaires on whether participants should or should not be able to choose to receive feedback of an IF ( Table 3: footnote 10), illustrating the complexities in interpreting opinions on this issue.

The findings of this study are of practical legal and ethical importance, and can be considered with regards to the duties of care, and the ethical principles of respect for autonomy, and beneficience and non-maleficence toward participants and towards the public. The legal and ethical background to UK Biobank’s approach was developed with input from its Imaging Working Group, its independent Ethics and Governance Council, representatives of its major funders (Wellcome Trust and the Medical Research Council), UK Biobank’s legal counsel and external legal counsel and ethics advice. In brief, it was considered likely that the duty of care owed to participants by radiographers would not be of a clinical standard, but rather what a reasonably competent radiographer conducting research imaging without clinical information could reasonable observe and report. This legal duty of care informs the ethical duties of radiographers, i.e., that they must be capable of meeting the standards of care which are detailed in the consent process. Therefore, in order to respect potential participants’ autonomy, it is paramount that UK Biobank have an IFs protocol in place, and that this protocol and its limitations are explained to and understood by participants. Our results reinforce the need for clarity in the information provided to participants about the feedback policy before they consent to imaging research studies. While participants’ understanding of what they had consented to was generally good, a substantial minority (around a quarter) incorrectly thought that they could choose whether or not to receive feedback. The information materials for the UK Biobank imaging study now further emphasize the difference between research and clinical diagnostic imaging, that the imaging is not a ‘health check,’ that not all serious disease will be detected, and that some potentially serious IFs will prove to be non-serious with further investigations ( http://imaging.ukbiobank.ac.uk/). Considering the ethical principles of beneficence and non-maleficence toward both participants and the public, our data suggest that feeding back potentially serious IFs which turn out not to be serious (false positives) can make some participants worse off, through exposure to the inconvenience, worry and potential harms of clinical assessments, including invasive procedures. Feedback of false positives also results in wider harm through the unnecessary use of publicly-funded health services. Missing a serious disease (false negative) does not make participants worse off compared to their status before receiving feedback of a potentially serious IF; rather, it fails to make participants better off. While the literature about IFs sometimes argues that feedback is inevitably beneficial ^21^, the balance of potential benefits and harms of earlier diagnosis (of IFs which are actually serious) is uncertain. It is important to reiterate that UK Biobank is a research resource which aims to facilitate research which will benefit public health, rather than provide any form of health services to individual participants. We therefore conclude that the responsibilities of researchers to avoid unnecessary harm to significant numbers of participants and disruption to publicly-funded health services mean that radiographer flagging (resulting in far fewer false positives while missing a small number of true positives with unclear benefit of earlier diagnosis) constitutes an ethically more justified approach in the UK Biobank imaging study than systematic radiologist review.

Some might argue that concerns about generating false positives suggest the case for a policy of no feedback of any IFs. However, in the light of legal advice regarding the duty of care it owed to participants as described above, UK Biobank decided not to withhold all feedback on potentially serious IFs, but to minimize the generation of false positives by only feeding back potentially serious IFs which are also confirmed by a radiologist. This approach to potentially serious IFs should be seen within the context of large-scale, population based imaging of healthy volunteers; a different approach may well be appropriate for other types of imaging studies, which may be smaller, based in clinical centres, have a different duty of care between research participants and researchers, or include participants with different characteristics (e.g., age) to those in the UK Biobank study.

While our underlying objective was to test the IFs protocol for the UK Biobank imaging study, our findings are of potential relevance in other contexts in which individuals are imaged prior to clinical presentation of disease, including public health and commercial screening. In both situations, it is important to consider the potential benefits of making a true positive diagnosis versus the potential harms to the individual and to publicly-funded health services, of a false positive diagnosis. The significant number of false positives generated by systematic radiologist reporting in our study implies that imaging of asymptomatic people should not be undertaken without appropriate concern for ensuring that the individuals being imaged do not end up worse off than they started.

### Strengths

Our study is the first to systematically follow up all participants receiving feedback about IFs and their GPs, giving the most comprehensive data on the impact of feedback of potentially serious IFs in any research imaging study to date and providing the first quantitative comparison of two different protocols for handling IFs. We have demonstrated for the first time the much lower rates of potentially serious IFs and, most importantly, false positives detected with a protocol in which radiologists report only those images which radiographers flag as having potentially serious IFs. Although the public support the principle of providing feedback of IFs ^14^, regardless of clinical severity ^12^, most previous studies did not survey people who had actually received feedback. Our findings are crucial to informing future policy surrounding feedback of IFs in research studies.

Our study was strengthened by good questionnaire response rates and near complete data on final diagnoses due to extensive efforts to gather these directly from participants and their GPs, and data collection at both early and later time periods following feedback. Results related to understanding of consent and impact of feedback on participants were confirmed and contextualised in a parallel, qualitative study.

### Limitations

Radiographer flagging rates could, in principle, have been influenced by a relative lack of experience with the first 1000 imaged participants, or by knowledge that radiologists were also reviewing all images. However, ongoing collection of data on potentially serious IFs in the 7000 participants imaged subsequently showed the prevalence of IFs detected by radiographers to be broadly consistent over time with a stable prevalence of potentially serious IFs confirmed by radiologists (mean proportion of 1.7%) ( Supplementary File 10).

Although questionnaire response rates by participants were generally high, only around two thirds of participants’ GPs responded about participants’ emotional well-being and overall net benefit/harm. The design of the questionnaires did not allow for quantification of the use of particular health services or evaluation of the associated costs. However, UK Biobank continues to collect data from participants with potentially serious IFs and their GPs through questionnaires, supplemented by linkages to national health datasets. This will enable further clinical, health economic and policy issues to be addressed using data from larger numbers of imaged participants.

Classification of final diagnoses as serious or not was based on clinical judgement of data available up to around six months following feedback of a potentially serious IF. Final diagnoses classified as serious may not actually shorten life span, or substantially impact on major body functions or quality of life in the 21 participants concerned, who were apparently healthy at the time of their imaging visits. Some potentially serious IFs may take longer than six months to diagnose, or for their full impact to become clear, potentially leading to an incomplete picture of the adverse impacts of feedback.

## Conclusions

The handling of potentially serious IFs merits serious consideration by researchers undertaking imaging research studies. Our data provide evidence to inform policy for large-scale research imaging in healthy populations, and are relevant to asymptomatic populations undergoing public health screening and commercial imaging. They demonstrate that systematic radiologist review of all images leads to the diagnosis of previously unknown serious disease in some participants. However, the great majority of these findings turn out not to be serious, resulting in unnecessary anxiety for the participant and unnecessary clinical assessment, which may include invasive procedures, provided by publicly-funded health services. Further, for those participants whose IFs do turn out to be serious, it is often difficult to ascertain whether this knowledge results in clear clinical benefit.

There is no ‘one-size-fits-all’ approach to handling IFs, as much depends on the purpose of the imaging, be that research, screening, or clinical care. In research studies of healthy volunteers, for whom there is no direct benefit for taking part, it is particularly critical to minimise harm. Based on these results, we suggest that this is achieved in an imaging study of UK Biobank’s scale and complexity with a protocol in which radiographers flag suspicious images for reporting by radiologists, rather than systematic review of all images by radiologists.

## Data availability

Due to the confidential nature of questionnaire responses and clinical information on participants with potentially serious incidental findings, it is not possible to publicly share all of the data on which our analyses were based, but extensive summaries of all relevant data are included in the supplementary material and within the linked online material.

Importantly, any bona fide researcher can apply to use the UK Biobank resource, with no preferential or exclusive access, for health related research that is in the public interest. Application for access to UK Biobank data involves registration and application via the UK Biobank website, with applications considered by the UK Biobank Access Sub-Committee. Following approval, researchers and their institutions sign a Material Transfer Agreement and pay modest access charges. Further information on applying to access UK Biobank data is available at: http://www.ukbiobank.ac.uk/register-apply/.

## Supplementary material

Supplementary File 1: Participant information leaflet. Consent for publication of the potentially identifying images was provided.

Supplementary File 2: UK Biobank magnetic resonance imaging parameters.

Supplementary File 3: UK Biobank lists of incidental findings.

Supplementary File 4: Example feedback letter sent to participants.

Supplementary File 5: Example feedback letter sent to general practitioners.

Supplementary File 6: Understanding of consent related to the feedback of potentially serious incidental findings: Data from 607 respondents from the first 1000 imaged UK Biobank participants.

Supplementary File 7: Final diagnoses of 217 potentially serious incidental findings.

Supplementary File 8: Invasive procedures performed to diagnose or treat potentially serious incidental findings.

Supplementary File 9: Clinical management (medication and procedures) of the 21 participants with potentially serious incidental findings which were finally diagnosed as serious.

Supplementary File 10: Rates of radiographer flagging and rates of radiologist confirmation of potentially serious incidental findings in the first 7000 imaged UK Biobank participants.
