# Supplementary material for: Impact of detecting potentially serious incidental findings during multi-modal imaging
Source: Wellcome Open Res. 2018 Aug 2;2:114. Originally published 2017 Nov 30. [Version 3] doi: 10.12688/wellcomeopenres.13181.3 (PMC6024231; doi:10.12688/wellcomeopenres.13181.3)
Supplement: Supplementary file 1 [file wellcomeopenres-2-16045-s0000.tgz › 15a62844-2c1d-4954-94f2-507b360840ab.pdf]

Supplementary File 1: Participant information leaflet

This is the version of the UK Biobank imaging study Participant Information Leaflet sent to the first 1000 imaged participants, and is no longer in use. It is available here for reference only.

The current version of the UK Biobank imaging study Participant information leaflet is available at http://imaging.ukbiobank.ac.uk/ under ‘Further documents.’

INFORMATION LEAFLET

Imaging Assessment Visit

UK Biobank is inviting you to take part in an important new study to help research. It involves taking pictures (scans) of your brain, heart, tissue and bones, so that scientists can study your internal organs in detail. This will help research into a wide range of diseases, including cancer, heart disease, dementia, diabetes, stroke and arthritis.

We aim to scan up to 100,000 people over the next few years. The scans, especially when combined with other health information you have provided to us, will create a health resource of global significance for many years to come. All of the information provided by you is stored in a confidential and secure manner. None of the data, samples and images provided to researchers will include personal identifying details.

You have been invited because you live within a reasonable travelling distance from the imaging assessment centre in Stockport. This invitation is not based on any other information that we have collected about you, either at your initial assessment or afterwards.

Taking part is entirely voluntary. Please take the time to read this leaflet carefully. It explains why we are asking you to help and what it would involve.

If anything is not clear, or if you would like more information, please telephone **0800-0-276-276 (free from most land lines) or 0292-0-765-597**, Monday-Saturday 8am-7pm, or email us at ukbiobank@ukbiobank.ac.uk

More information about UK Biobank is available at www.ukbiobank.ac.uk. There will also be further opportunities to ask questions when you arrive at the imaging assessment centre.

Thank you for your continued support of UK Biobank.

Improving the health of future generations

What is UK Biobank?

UK Biobank is a large, publicly-funded resource to help scientists from around the world to improve the prevention, diagnosis and treatment of a wide range of illnesses (such as cancer, heart disease, diabetes, dementia, and joint problems). Its goal is to improve the health of future generations.

UK Biobank allows scientists to study how health is affected by lifestyle, environment and genes. By studying the answers, measurements and samples collected from the 500,000 participants, researchers will be able to work out why some people develop particular diseases while others do not.

This research will help to find new ways to prevent premature death and disability. While taking part in UK Biobank is not intended to help participants directly, it should give future generations a much better chance of living their lives free of diseases that disable and kill.

Why does UK Biobank want to scan me?

Taking pictures of organs inside the body (such as the brain and heart) as well as the surrounding tissues and bones will allow scientists to study how the structure and function of the body's organs are related to the development of disease. The combination of these pictures with other information already collected about you will provide a substantial amount of new and important data for health research on a wide range of diseases.

Why have I been invited?

Participants who live within a reasonable distance from the imaging assessment centre are being invited to take part. The centre is at the UK Biobank's Co-ordinating Centre in Stockport. The cost of setting up such a centre means it is not possible to open one in every place in which UK Biobank undertook its original assessments. This means that some people may have to travel further to participate, although we will cover your travel expenses.

Invitations are not based on other information that has already been collected about you.

biobank uk
Improving the health of future generations

What scans will be performed and why?

Magnetic resonance imaging (MRI): This type of scan uses painless magnetic waves to take detailed pictures of the inside of the body (such as organs, tissues and bones). We would like to take two scans: one of the brain and the other of the heart and of the body (mainly covering the abdomen). The scanners are similar to those used in the NHS, except for being a little wider so that people are as comfortable as possible.

- ***Brain MRI scan.*** This will provide information about the structure and function of the brain. It will enable us to obtain information on, for example, which parts of the brain are important for carrying out certain tasks and how different parts of the brain are connected.

- ***Heart and body MRI scan.*** This will provide information on the size of the heart chambers and blood vessels, and changes in heart size as it beats. It will also provide detailed information on the amount and distribution of fat in the body.

Neck artery ultrasound scan. This scan uses ultrasound (high-frequency sound waves) to produce pictures of the blood vessels on either side of the neck. They will help scientists study the build-up of fatty substances (like cholesterol) in these major blood vessels.

Dual-Energy X-ray Absorptiometry (DXA) scan. This scan uses low-energy x-rays to provide a precise measure of bone density throughout the body. Detailed pictures of the spine, hips and knees will help scientists studying diseases like arthritis.

Am I eligible to take part?

All of these scans are safe and painless, and are similar to those used routinely in the NHS. However, since MRI scans involve the use of a magnet, you will not be able to take part if you have any metal or electrical implant, or if you have had an accident where metal may have entered your body.

If you have had recent surgery, you will be able to take part but your visit will not be able to occur until at least six weeks after your operation. You will also not be able to take part if you have medical problems that make it difficult to conduct the scans (e.g., severe hearing or breathing problems, tremors, etc.).

Do I have to take part in this imaging assessment?

No; attendance is entirely voluntary. We do understand that you may not have time or be able to help on this occasion.

What should I do if I am interested in attending?

Please let us know as soon as possible if you are willing to attend by telephoning us on:

0800-0-276-276 (free from most land lines) or 0292-0-765-597,
Monday to Saturday, 8am - 7pm

During this call, you will be asked a series of questions to find out whether you are eligible to help. Please visit our website (www.ukbiobank.ac.uk) to see the questions that will be asked, but please do not worry if there are technical terms used that you do not understand as these will be explained to you during the call. If you are willing to participate, you will also be able to ask questions about the assessment visit.

If you are eligible, you will be able to arrange an appointment during this call. Appointments are generally available from 8am to 6pm seven days a week to help find a convenient time for you.

We will then send you a letter confirming your appointment, along with detailed directions to the assessment centre. We will also send you an email or text reminder a couple of days before your planned visit.

Can I claim travel expenses for attending?

Yes, please do claim back any reasonable travel expenses (including standard train and bus fares, and mileage for car, motor cycle or bicycle journeys). A claim form will be handed to you at the end of your visit. It would help us if you attached your travel receipts.

There is ample free parking space at the centre. It is also within easy access of Stockport train station, where taxis (paid for by UK Biobank) are available. If you are registered as disabled, you can also claim travel expenses for a companion.

How do I prepare for the imaging assessment visit?

When you come for your appointment, please:

- Bring with you the confirmation letter and travel directions, so you have no difficulty in finding us.
- Bring any reading glasses that you use since you have to be able to read clearly from a computer screen (as at your original assessment visit).
- Bring along your glasses prescription (if you have it), since we will need to provide you with specially adapted glasses that match your usual prescription for the brain MRI scan.
- Bring details of the name and address of your doctor (GP).
- Remove any jewellery that can be easily removed (although we will secure with removable tape any jewellery, such as wedding rings, that cannot be removed). Please ensure that your underpants do not contain metal fastenings.

- Remove any hair grips and makeup as these may contain metallic fragments which could cause a heating sensation during the MRI scans.

- Remove any skin patches (often used for hormone-replacement therapy [HRT], nicotine replacement, pain relief, contraception, glyceryl trinitrate [GTN], etc.) as these may also contain metallic components. We recommend that you bring a spare patch with you to put on after the scans.

- Be prepared to spend about 4 hours at the assessment centre. Refreshments (such as salads, sandwiches, tea, coffee and soft drinks) will be provided during the visit. (There is no need to avoid eating before your visit.)

What happens DURING the imaging assessment visit?

The assessment will take about 4 hours. It will involve the following (in this order):

Initial steps

- A trained member of staff will ask the same questions you answered when you made the appointment. This is to double check that you are able to undergo all of the different types of scan. (If you cannot have some particular scans, then you would still be able to take part in the rest of the visit).

- You can ask any questions that you might have, and will then be asked to sign a consent form. This tells us that you agree to be scanned, and that you understand the process and the implications.

- You will be shown to a private cubicle where you will be given special, loose-fitting clothes to change in to. You will not need to remove underpants, but women will be asked to remove their bras, since they may contain metal.

- You will be asked to leave any loose metal objects (such as money, credit cards, keys, pens, mobile phone, jewellery, watches, hair pins, metal dentures, hearing aids and spectacles), as well as any skin patches (e.g., nicotine or other replacement therapy), in one of the secure lockers.

- Men may be asked if a staff member can shave a small section of their chest hair. This is so that electrical leads attached to sticky pads can be placed on the skin for the electrocardiogram (ECG; an electrical recording of the heart) and the heart MRI scan.

MRI scans

- The brain and heart/body MRI scans each take about 30 minutes.

- You will be shown into a room that houses one of the two MRI scanners. The scanner is a large cylinder with a tube running through the middle which is open at both ends (see picture below). You will be asked to lie down on a comfortably padded table that gently glides you into the scanning tube. Depending on the part of your body being scanned, you

will be moved into the scanner either head first or feet first.

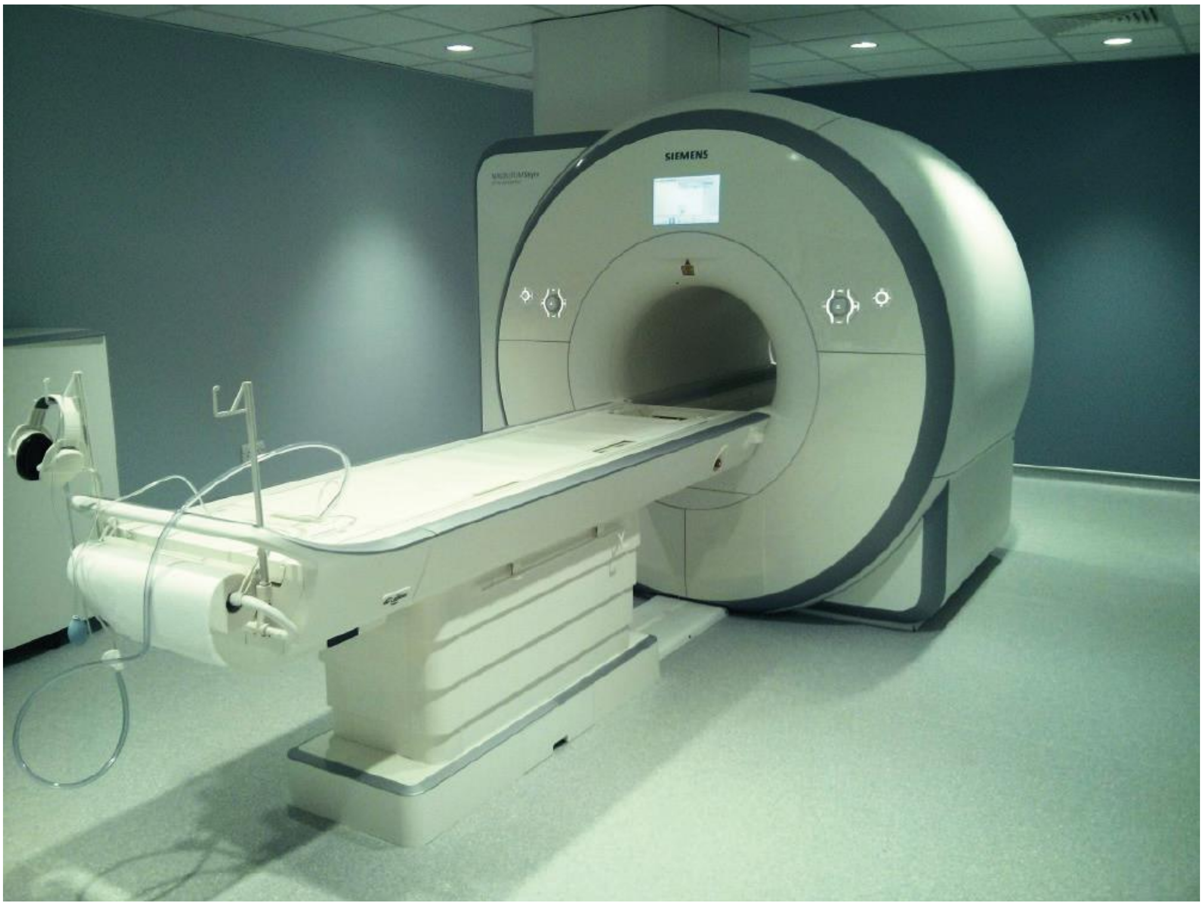

A magnetic resonance imaging (MRI) scanner

- The MRI scanner is controlled by a computer which is in a different room. A specially trained technician will operate the computer. They will be able to see you through a window throughout the scan, and you will be able to talk to them through an intercom.

- MRI scanners can be noisy. We will provide you with headphones to protect your ears, but you will still be able to talk to the operator throughout the scan. You will be given a hand-held buzzer so that you can stop the process at any time if you wish.

- During the MRI scans, you will be asked to do things. For example, you will be shown something on a screen during the brain scan, and you will be asked to hold your breath for a short period of time during the heart scan.

Neck ultrasound scan

- The neck scan takes about 10 minutes.

- You will be asked to lie face-up on a firm table. A clear water-based gel will be applied to your neck. A hand-held probe will then be placed against your skin and moved up and down your neck (see picture below).

- You will be asked to tilt or turn your head as the probe is swept over the entire length of both sides of your neck.

- The probe only covers your neck and does not touch your face or other parts of your body.

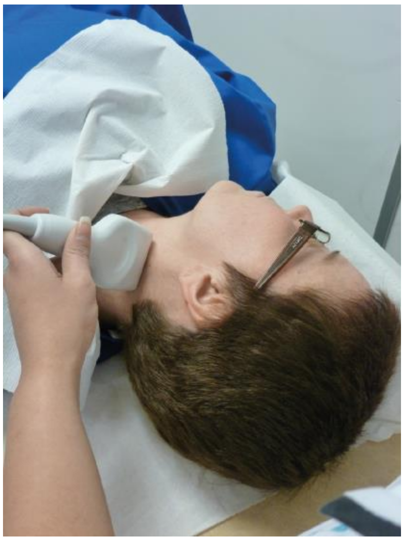

Neck ultrasound

DXA scan

- The DXA scan takes about 20 minutes.

- You will be asked to lie on a firm table while an arm passes over you (see picture below) to take X-ray pictures of your bones. You will be asked to lie in various positions so that the scanner can take pictures of different parts of your body.

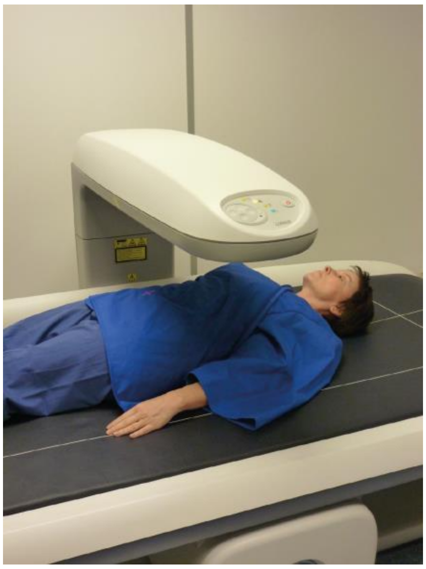

DXA scanner

Other assessments

While you are with us, we would also like to take some more samples and repeat a number of measurements that we did at your first visit to UK Biobank several years ago. This information will allow scientists to take account of any changes in health and lifestyle over that time.

We will ask you to:

- Give another small sample of blood (about 3 tablespoons), saliva and urine for long-term storage and analysis.
- Answer again questions on your health, lifestyle and diet, memory, work and family history.
- Have repeat measurements of your blood pressure, pulse rate, height, weight, body fat, grip strength, heel bone density and lung function. You will also have an electrocardiogram (ECG) to measure the electrical activity of your heart.

What are the possible BENEFITS of taking part?

There are no direct benefits to you in taking part. However, the information about you from the imaging and other assessments will be valuable for helping scientists to better understand how a wide variety of diseases develop and to find new ways to prevent them.

What are the possible RISKS of taking part?

Undertaking the imaging assessment visit should not cause you any harm. We have chosen to perform scans and other physical measures that are safe, painless, relatively quick and comfortable.

The MRI scans use powerful magnets and great care is taken to prevent magnetic objects from entering the MRI room. Before you enter, we will ask you questions so that special precautions can be taken, if needed.

MRI scans involve lying flat in a slightly confined space and a small number of people may find this uncomfortable. However, the space in the scanners used in this assessment is wider (at least 70 cm, or 27 inches, in diameter) than in those typically used in hospitals, to ensure that participants are as comfortable as possible.

The low energy DXA scan involves a small dose of radiation (approximately 20 µSv units). This is the same amount as a standard chest X-ray or about one week’s worth of natural background X-rays. By comparison, one trans-Atlantic flight exposes you to about four times as much radiation as that from a DXA scan.

You may feel some discomfort when you have blood taken, although our staff are specially trained to reduce this discomfort.

Do I need to have all the measurements?

When you call to make your appointment, we will check whether you are eligible to be scanned. This is because we do need your agreement to take part in all of the imaging scans before you make an appointment.

If it turns out when you arrive at the imaging assessment centre that you are no longer eligible for some of the scans, then you will still be able to take part in the rest of the assessment.

You do not have to have all the physical measures or to give a blood, urine or saliva sample if you don't want to. Similarly, if you feel uncomfortable about answering certain questions then you do not need to answer them.

Do I get any routine results from the visit?

As was the case at your initial assessment, you will receive information at the end of the visit about some of the physical measurements made during the assessment (blood pressure, weight, body mass index, waist circumference, percent body fat, heel bone ultrasound and lung function).

However, you will not receive any other routine results or pictures from the scans. It is important for you to be aware that this visit is not a clinical appointment or a 'health check'. You should, therefore, not rely on the absence of feedback from us as any form of reassurance regarding your health.

What will I be told if something suspicious is seen during my scans?

Imaging scans often show abnormalities, but most of these are no cause for concern.

The scans being performed in UK Biobank are not intended to diagnose an illness. They are not designed to identify any particular abnormalities and will not be routinely analysed by doctors or other specialists. Instead, they are being taken and stored for future research.

The technicians conducting the scans will be looking at the images to ensure their quality. It is important to understand that they will not be looking at them to identify particular health problems.

However, if a technician does notice something unusual that they think might be serious they will refer that scan to a specialist doctor for review. Something would be considered potentially serious if it indicated the possibility of a condition which, if confirmed, would carry a real prospect of seriously threatening life span, or of having a substantial impact on major body functions or quality of life.

If the specialist doctor agrees that the abnormality may be serious (regardless of whether or not it can be treated), then we will write to you and your GP (usually within two weeks of your visit).

For example, you and your GP would be informed if we saw an abnormality on one of your scans that looked like a tumour. However, we would not inform you if we saw an abnormality that looked like gallstones or a simple cyst, as such findings are common in healthy people

and not considered serious. Please see our website (www.ukbiobank.ac.uk) for further information about the types of potentially serious findings that we would inform you and your GP about if they are noticed during the scans and confirmed by a specialist doctor.

We estimate that about 10 to 15% of participants may have an abnormality considered to be potentially serious, but we will not know this number for sure until the project gets fully underway.

The reporting processes set in place will be carefully monitored, with ongoing training provided for the technicians doing the scanning.

What may happen if I am told about something suspicious on my scans?

The identification of an abnormality may mean that your GP refers you to specialists for further investigation and treatments. While some abnormalities may result in the diagnosis of a serious untreatable condition, others may turn out to be of little or no concern.

For some conditions, having an earlier diagnosis can be beneficial. But for other abnormalities detected on scans, knowing about them many years in advance can lead to unnecessary anxiety, investigations and treatments. The identification of some abnormalities could affect your ability to drive or work and could also make it difficult for you to obtain future travel, health or life insurance. It is important for you to understand that if we do not contact you and your GP it does not necessarily mean that you do not have any abnormalities. It simply means that no such abnormality was noticed by the technicians taking the scans.

It is also important to note that you can only take part in the imaging study if you feel able to consent to both you and your GP being informed if a potentially serious abnormality is noticed on one of your scans. If you feel that, in your case, the potential anxiety and uncertainty of being told about a possible serious abnormality, or the inconvenience and disruption to your life caused by further investigations, is likely to outweigh any benefit to you, we would quite understand if you decide not to take part in the imaging study.

How are we going to assess the impact of telling participants about potentially serious abnormalities?

For participants who are told of a potentially serious abnormality, we wish to find out how this has affected them, their family and friends, and the people involved in their care in the NHS. Very little is known about the impact of receiving information like this and this research would allow us to improve what we do and help others doing similar research.

We will, therefore, contact those participants 6 weeks and 6 months after providing such feedback to find out what, if anything, happened as a result of receiving it. We will also contact their GP about 6 months after writing to them to find out about any investigations and treatments.

Who will be able to use my information and samples?

Data and samples from your visit (scans, blood results and other data) will be stored for many years to come. The information will be used by approved researchers for medical and other

health-related research. This includes researchers who are working in other countries and in commercial companies looking for new treatments. Scientists will have to obtain scientific and, if necessary, ethics approval.

Results from all of these studies will be put back into the UK Biobank database for other researchers to use. Scientists must also publish the results of all research based on the UK Biobank resource so that everyone can benefit from it. Details of research that is being done using the resource is available on the website (www.ukbiobank.ac.uk).

We will never forward any individual's information, samples or test results to insurance companies or employers. Neither will we allow access to the police, security services, relatives or lawyers, unless forced to do so by the courts.

Published research that uses the UK Biobank resource will be made available to participants, and anyone else who might be interested, at www.ukbiobank.ac.uk.

How will information about me be kept confidential?

UK Biobank has put a number of rigorous measures in place to protect the confidentiality of participants. These include:

- No personal identifying details are included with data or samples provided to researchers.
- Information that might identify individuals (such as their names and addresses) is kept separately from other information about them in UK Biobank's databases.
- High level computer security is used to block unauthorised access (for example, by "hackers") to the computers that hold personal information.
- Access to personal information is restricted as much as possible, and all staff working on UK Biobank sign confidentiality agreements as part of their employment contracts.

Who do I contact if I have any questions?

If this leaflet does not answer your questions, please telephone us on 0800-0-276-276 (free from most land lines) or 0292-0-765-597, Monday-Saturday 8am-7pm for more information, or visit our website at www.ukbiobank.ac.uk.

If you would like to contact the person in charge, please send a letter or email to:

Professor Sir Rory Collins

UK Biobank
1-2 Spectrum Way
Adswood
Stockport
Cheshire
SK3 0SA

Email: ukbiobank@ukbiobank.ac.uk

We shall reply to your letter promptly in writing, unless you enclose your telephone number and wish to discuss your concerns with us.

UK Biobank Limited (company no. 4978912) is a registered charity in England & Wales (1101332) and in Scotland (SCO39230)

Version January 2014
