# Supplementary material for: Impact of detecting potentially serious incidental findings during multi-modal imaging
Source: Wellcome Open Res. 2018 Aug 2;2:114. Originally published 2017 Nov 30. [Version 3] doi: 10.12688/wellcomeopenres.13181.3 (PMC6024231; doi:10.12688/wellcomeopenres.13181.3)
Supplement: Supplementary file 2 [file wellcomeopenres-2-16045-s0001.tgz › 623795ce-3e6a-46e1-9bec-5278c799c7ed.pdf]

Supplementary File 2: UK Biobank magnetic resonance imaging parameters

Region and sequence	Voxel dimensions (mm)	TR (ms)	TE (ms)	N slices	Other parameters
**Brain MRI[1]**					
T1	1 x 1x 1	2000	-	208	TI 880 ms
FLAIR	1.05 x 1 x 1	5000	395	192	TI 1800 ms
SWI+T2*	0.8 x 0.8 x 3	27	9.4, 27[2]	48	-
Functional MRI: Rest	2.4 x 2.4 x 2.4	735	39	64	MB 8, 490 volumes
Functional MRI: Task[3]	2.4 x 2.4 x 2.4	735	39	64	MB 8, 332 volumes
Diffusion[4]	2.0 x 2.0 x 2.0	3600	92	72	MB 3
**Cardiac MRI**					
Long axis cine	1.8 x 1.8 x 6	32.64	1.16	-	373 ms resolution
Short axis truFISP cine	1.8 x 1.8 x 8	31.56	1.10	-	373 ms resolution
ShMOLLI	0.9 x 0.9[5] x 8	368.28	1.07	1	TI 0.1-5 s
Cine, single breath hold tagging	1.4 x 1.4 x 8	41.05	3.90	-	Grid tag, 3 short axis views
SSFP cine LV outflow tract and aorta	1.8 x 1.8 x 6	32.64	1.16	-	373 ms resolution
Flow sensitive cine aorta	1.8 x 1.8 x 6	37.12	2.47	-	TI 1.0 ms, 373 ms resolution
**Body MRI**					
T1 abdomen	1.9 x 1.3 x 10	450	11	12	-
3D-Dixon water fat separation neck to knees	2.2 x 1.2 x 10	3.23	1.44	-	Coronal and axial planes
T1 pancreas	1.2 x 1.2 x 1.12	2.95	1.12	80	-
Single breath hold liver and pancreas multi-echo	2.2 x 2.2 x 10	150	1.23-14.76[6]	-	-

mm = millimetres, TR = repetition time, ms = milliseconds, TE = echo time, MRI = magnetic resonance imaging, TI = inversion time, FLAIR = fluid attenuation inversion recovery, SWI = susceptibility weighted imaging, MB = multiband pulses, FISP = fast imaging with steady-state free precession, ShMOLLI = shortened modified Look-Locker inversion recovery, SSFP = steady state free precession, LV = left ventricle

- = Not applicable

[1] Participants imaged before August 18th 2014 (615/1000) also had a T2-weighted brain MRI sequence, however this was removed from the imaging protocol after this date and is no longer included in the UK Biobank imaging study. Additional modifications were made to the imaging protocol over the period during which the first 1000 participants were scanned, but these modifications were unlikely to affect the detectability or characterisation of potentially serious incidental findings and are not detailed here.

[2] Two echoes required

[3] Harari emotional faces task

[4] b=1000 and b=2000 s/mm^2, 50 directions per shell

[5] Interpolated

[6] 12 gradient recalled echoes
