# Supplementary material for: Impact of detecting potentially serious incidental findings during multi-modal imaging
Source: Wellcome Open Res. 2018 Aug 2;2:114. Originally published 2017 Nov 30. [Version 3] doi: 10.12688/wellcomeopenres.13181.3 (PMC6024231; doi:10.12688/wellcomeopenres.13181.3)
Supplement: Supplementary file 3 [file wellcomeopenres-2-16045-s0002.tgz › 42a50600-9749-4100-909a-15d69d64ce30.pdf]

Supplementary File 3: UK Biobank lists of incidental findings

UK Biobank developed lists of findings which would be considered potentially serious, and findings not considered serious for use by radiographers and reporting radiologists. These lists were based on lists generated by the German National Cohort, and are subject to ongoing review.

Table i: Incidental findings on brain MRI

**Potentially serious for feedback**	**Not for feedback**
Acute brain infarction	Asymmetrical ventricles
Acute hydrocephalus	Chiari malformation[4]
Acute intracranial haemorrhage[1]	Chronic hydrocephalus
Arachnoid cyst[3]	Developmental anomalies (including venous anomalies)
Colloid cyst of third ventricle	Lipoma of corpus callosum
Intracranial mass lesion[2]	Non-acute brain infarction
Mastoiditis	Non-specific white matter hyperintensities
Suspected intracranial aneurysm or vascular malformation	Regional or global atrophy
	Suspected demyelination

[1] Not old bleeds, or microbleeds only detected on gradient recalled echo sequences

[2] Except meningiomata in locations considered highly unlikely to cause problems

[3] Only if large and considered likely to increase the risk of developing a subdural haematoma

[4] Descent of part of the cerebellum +/- brainstem below the foramen magnum

Table ii: Incidental findings on cardiac MRI

Potentially serious for feedback	Not for feedback
Aortic dissection	Atelectasis
Cardiac mass (including thrombus)	Calcified pleural plaque
Central pulmonary embolus	Calcified pulmonary nodule
Haemodynamically relevant pericardial effusion >2 cm	Emphysema
Heart valve defects[1]	Right sided descending aorta
Hilar, mediastinal, axillary or cervical lymphadenopathy[2]	
Lobar pneumonia or lung consolidation	
Lung mass > 2 cm	
Mediastinal mass > 2 cm	
Pleural effusion	
Pleural mass > 2 cm	
Pneumothorax	
Severe left or right ventricular dilation or dysfunction	
Severe left ventricular hypertrophy > 2 cm thick wall	
Thoracic aortic aneurysm > 5 cm	

[1] Severe regurgitation jet of any valve or severe turbulence (suggesting valve stenosis)

[2] >1.5 cm and >3 lymph nodes grouped in a circumscribed region

Table iii: Incidental findings on the abdominal portion of the body MRI

Potentially serious for feedback	Not for feedback
Abdominal aortic aneurysm > 5 cm	Abdominal wall hernia
Acute exudative pancreatitis	Bladder diverticulum
Adrenal lesion > 2 cm	Chronic cholecystitis
Ascites	Chronic pancreatitis
Cholestasis (intra- or extra-hepatic)[1]	Fatty liver
Deep vein thrombosis	Fibroids
Hepatomegaly	Gallstones
Ileus	Hiatus hernia
Intra-abdominal mass > 3 cm	Left sided inferior vena cava
Irregular/nodular liver margin	Liver cyst
Lymphadenopathy[2]	Renal calculus
Multiple small non-cystic, liver lesions (non haemangioma-like)	Simple renal cyst
Pneumoperitoneum	Single kidney
Portal vein occlusion	
Pyelonephritis	
Renal artery stenosis > 80% or bilateral	
Solid / cystic pancreatic tumour	
Solid gallbladder lesion	
Solid liver lesion	
Solid/semi-solid renal tumour > 2 cm	
Spleen infarction	
Splenomegaly > 15 cm	
Urinary obstruction	
Urinary tract mass > 2 cm	

[1] Common bile duct >15 mm (or >20 mm post-cholecystectomy)

[2] >1.5 cm and >3 lymph nodes grouped in a circumscribed region

Table iv: Incidental findings on dual energy X-ray absorptiometry

Potentially serious for feedback	Not for feedback
Major vertebral fracture	Non-skeletal findings
Primary skeletal malignancy	
Skeletal metastases	

Carotid Doppler ultrasound

Although asymptomatic carotid stenosis may be picked up by carotid ultrasound, its relevance in predicting prognosis over and above conventional vascular risk factors is not established, and so it was not considered to be a potentially serious incidental finding. Extra-carotid findings were not considered relevant for UK Biobank's imaging study as the radiographers conducting the imaging are specifically trained in the vascular component of this imaging modality only. Hence, carotid Doppler data do not form part of this manuscript.
