# Supplementary material for: Impact of detecting potentially serious incidental findings during multi-modal imaging
Source: Wellcome Open Res. 2018 Aug 2;2:114. Originally published 2017 Nov 30. [Version 3] doi: 10.12688/wellcomeopenres.13181.3 (PMC6024231; doi:10.12688/wellcomeopenres.13181.3)
Supplement: Supplementary file 4 [file wellcomeopenres-2-16045-s0003.tgz › 32a024f9-3c77-418b-8620-a17b14895c39.pdf]

Supplementary File 4: Example feedback letter sent to participants

Date

«participant_name»

«participant_address»

Dear «participant_name»,

Invitation to make an appointment with your GP following your visit to the UK Biobank imaging assessment visit

Thank you for your recent attendance at the UK Biobank imaging assessment visit.

We are writing to inform you that, during the scanning process, something was noticed on your [name_of] scan that your GP may want to follow up. We have informed «practice_name» of this possible abnormality, and recommend that you make an appointment to see your GP at your earliest convenience.

If these GP details are incorrect, please let us know as soon as possible by telephoning the Participant Resource Centre on 0800-0-276-276 (free from most land lines) or 0292-0-765-597, Monday-Saturday 8am to 7pm, or by emailing imaging.queries@ukbiobank.ac.uk.

Please do not be unduly alarmed by this letter. Whilst we aim to inform you only of abnormalities that might be potentially serious, it is still likely that many of these findings will turn out not to be of concern, or be something of which you are already aware. As indicated to you at the time of your assessment visit, the scans taken by UK Biobank are not specifically designed to detect clinical abnormalities, and so cannot generally be used to determine exactly what a possible abnormality is.

Your GP will be able to advise you as to what further action or investigations, if any, are needed.

Yours sincerely,

Professor Sir Rory Collins

UK Biobank Principal Investigator, and

Professor of Medicine & Epidemiology,

University of Oxford.
