# Supplementary material for: Impact of detecting potentially serious incidental findings during multi-modal imaging
Source: Wellcome Open Res. 2018 Aug 2;2:114. Originally published 2017 Nov 30. [Version 3] doi: 10.12688/wellcomeopenres.13181.3 (PMC6024231; doi:10.12688/wellcomeopenres.13181.3)
Supplement: Supplementary file 5 [file wellcomeopenres-2-16045-s0004.tgz › 6ed59666-815f-482b-8993-f0ff371d5a31.pdf]

Supplementary File 5: Example feedback letter sent to general practitioners

Date

«GP_name»

«practice_address»

Dear «GP_name»

Cc «partipant_name»; «nhs_number»

Report of a potentially serious abnormality on an imaging scan from UK Biobank

«partipant_name»; «nhs_number» recently attended an imaging assessment visit as part of their participation in UK Biobank. UK Biobank is a population-based cohort study, established by the Medical Research Council and Wellcome Trust with the support of the Department of Health, which recruited over 500,000 middle-aged people during 2006 to 2010, some of whom are now participating in our imaging sub-study. This involved undergoing brain, heart and abdominal MRI scans, a carotid ultrasound scan and a DXA low energy X-ray scan, as well as answering questions, having non-imaging measurements and providing biological samples.

The imaging scans taken by UK Biobank are intended for research use only; they are not optimised for identifying any particular clinical abnormalities and may not provide sufficient information for diagnostic purposes. However, the radiologist/specialist who reviewed «participant_name»'s [name of] scan reported an incidental finding that may be potentially serious (i.e. indicating the possibility of a condition which, if confirmed, carries a real prospect of threatening life span, or of having a substantial impact on major body functions or quality of life).

A copy of the specialist's report is provided with this letter in case you feel further referral or clinical investigation is warranted. If required, you can request a digital copy of the relevant scans by emailing imaging.queries@ukbiobank.ac.uk or by telephoning Mr. Steve Garratt on 0161 475 5378. Alternatively you can write to us at the address shown below.

We have informed «participant_name» that a possible abnormality was found (see attached letter) on one of their scans and advised them to make an appointment to see you at their earliest convenience.

There is little consensus in the UK (or elsewhere) on the balance of benefit versus harm in telling research participants about incidental findings from imaging studies. In order to improve our procedures and those of other research projects in the future, we will contact you again in a few months with a short questionnaire asking you about the impact on the health service of providing this information.

We would be grateful if you could let us know as soon as possible if «participant_name» is not registered with your practice. Many thanks for your help.

Yours sincerely,

Professor Sir Rory Collins,

UK Biobank Principal Investigator, Prof. of Medicine & Epidemiology, University of Oxford.
