# Supplementary material for: Impact of detecting potentially serious incidental findings during multi-modal imaging
Source: Wellcome Open Res. 2018 Aug 2;2:114. Originally published 2017 Nov 30. [Version 3] doi: 10.12688/wellcomeopenres.13181.3 (PMC6024231; doi:10.12688/wellcomeopenres.13181.3)
Supplement: Supplementary file 6 [file wellcomeopenres-2-16045-s0005.tgz › 4f46da3d-dcae-4b45-8bd5-65aa3a61e05e.pdf]

Supplementary File 6: Understanding of consent related to the feedback of potentially serious incidental findings: data from 607 respondents from the first 1000 imaged UK Biobank participants

	Response[1]					
	Correct		Incorrect		Don't know	
**As far as you are concerned, when you consented to participate, which of the following did you agree to?**	**n/N**	**%**	**n/N**	**%**	**n/N**	**%**
My imaging scans and results would be given to me at the end of the visit[2]	526/607	86.7	49/607	8.1	32/607	5.3
In the event a potentially serious finding was identified on a scan:						
I could choose whether my GP and I would be informed[2]	381/607	62.8	158/607	26.0	68/607	11.2
Both my GP and I would automatically be contacted[3]	454/607	74.8	119/607	19.6	34/607	5.6
I would be told about this finding during the assessment visit[2]	540/607	89.0	19/607	3.1	48/607	7.9
I would be told about this finding after the assessment visit[3]	251/607	41.4	300/607	49.4	56/607	9.2

[1] Proportions of participants answering each question correctly, incorrectly or answering that they did not know were similar irrespective of whether or not participants had a potentially serious incidental finding (IF), and irrespective of whether any potentially serious IF was finally diagnosed as clinically serious or non-serious.

[2] The correct response was 'no'.

[3] The correct response was 'yes'. However, in retrospect these questions were deemed ambiguous. The participant information leaflet described the UK Biobank IF policy, including that a finding identified on a scan by a radiographer would only be fed if confirmed by a radiologist. Taking this in to account, if participants considered the case where a finding identified by a radiographer was not then confirmed by a radiologist, some participants may reasonably have concluded that they would not automatically be contacted about a finding identified on a scan, or that they might always not be told about a finding after the assessment visit.
