# Supplementary material for: Impact of detecting potentially serious incidental findings during multi-modal imaging
Source: Wellcome Open Res. 2018 Aug 2;2:114. Originally published 2017 Nov 30. [Version 3] doi: 10.12688/wellcomeopenres.13181.3 (PMC6024231; doi:10.12688/wellcomeopenres.13181.3)
Supplement: Supplementary file 7 [file wellcomeopenres-2-16045-s0006.tgz › 0e4468c4-7c06-4bf0-8ffb-488e7894937d.pdf]

Supplementary File 7: Final diagnoses of 217 potentially serious incidental findings

Image modality	Final diagnosis	Number of scans identified by radiographer flagging	Number of scans identified by systematic radiologist review
	**Serious final diagnoses**		
Brain MRI	Pituitary tumour	0	2
	Arachnoid cyst with hydrocephalus	1	1
	Meningioma compressing brainstem	1	1
Cardiac MRI	Thoracic aortic aneurysm[1]	3	5
	Lung tumour	0	3
	Cardiomyopathy	0	2
	Atrial fibrillation	0	1
	Coronary heart disease	0	1
	Heart block and LV impairment	0	1
Body MRI: Abdomen	Abdominal aortic aneurysm > 5 cm	0	1
	Gastrointestinal stromal tumour	0	1
	Pancreatic neuroendocrine tumour	0	1
DXA	Osteoporotic crush fracture	0	1
**All modalities: serious final diagnoses**		5	21
	**Non-serious final diagnoses**		
Brain MRI	Benign cyst/lesion	2	15
	Already known diagnosis	0	1
	Suspected lesion not confirmed	0	3
Cardiac MRI	Lung diagnosis – not serious	2	28
	Suspected lesion not confirmed	0	18
	Other non-serious diagnosis	0	10
	Cardiac diagnosis – not serious	0	8

	Already known cardiac diagnosis	0	7
	Already known lung diagnosis	0	2
Body MRI: Abdomen	Benign lesion (e.g. cyst)	6	57
	Suspected lesion not confirmed	0	13
	Already known diagnosis	1	4
	Other non-serious diagnosis	0	4
	Abdominal aortic aneurysm < 5 cm	1	2
Body MRI: Leg	Bone/soft tissue diagnosis – not serious	0	5
	Suspected lesion not confirmed	0	2
	Already known finding	0	1
DXA	Already known diagnosis	1	5
	Non-serious diagnosis	0	5
	Suspected lesion not confirmed	0	2
**All modalities: non-serious final diagnoses**		13	192
	**Uncertain final diagnoses**		
Cardiac MRI	Lung nodule, unclear nature	0	2
	Lung consolidation, unclear nature	0	1
DXA	Crush fracture T11, unclear relevance	0	1
**All modalities: uncertain final diagnoses**		0	4

[1] One participant with a thoracic aortic aneurysm was also found to have an atrial myxoma, which was resected at the time of aneurysm repair.

[2] Four findings could not be classed as serious or not serious by April 2016**:** one participant with a lung nodule was still under follow-up; another participant with a lung nodule underwent follow-up and was found to have lymphoma, but it was unclear whether the nodule was related to the lymphoma or not; one participant with lung consolidation reported that the final diagnosis may be scarring or bronchoalveolar cell carcinoma (this participant was also noted to have a meningioma compressing their brainstem on brain MRI, so that their overall final diagnosis clinical severity was classified as serious); we were unable to contact one participant with a crush fracture to determine the grade of the fracture, or whether they had been diagnosed with or treated for osteoporosis.
