# Supplementary material for: Impact of detecting potentially serious incidental findings during multi-modal imaging
Source: Wellcome Open Res. 2018 Aug 2;2:114. Originally published 2017 Nov 30. [Version 3] doi: 10.12688/wellcomeopenres.13181.3 (PMC6024231; doi:10.12688/wellcomeopenres.13181.3)
Supplement: Supplementary file 8 [file wellcomeopenres-2-16045-s0007.tgz › 90084f4d-116d-4cd8-a5b0-3e0439031479.pdf]

Supplementary File 8: Invasive procedures performed to diagnose or treat potentially serious incidental findings

Final diagnosis	n participants in diagnostic category	n/N with data available who underwent an invasive procedure[1]	Modality	Invasive procedure (one participant per procedure unless otherwise indicated)
Serious	21	11/18 (61.1%)	Brain MRI	Third-ventriculostomy and fenestration of arachnoid cyst<br>Resection of meningioma compressing brainstem<br>Transphenoidal resection of large pituitary macroadenoma
			Cardiac MRI	Biopsy and resection of pulmonary nodule<br>Coronary angiogram to investigate poor LV function (n=2)<br>Resection of lung cancer<br>Repair of thoracic aneurysm (n=2)
			Body MRI: Abdomen	EUS FNA and Whipple procedure for pancreatic neuroendocrine tumour<br>Resection of gastrointestinal stromal tumour
Non-serious or uncertain	158	12/144 (8.3%)	Cardiac MRI	Aspiration of breast cyst<br>Bronchoscopy to investigate lung findings<br>FNA of thyroid<br>Resection of pulmonary nodule
			Body MRI: Abdomen	Colonoscopy to investigate thickened sigmoid (n=2)<br>Hysterectomy for ovarian cysts<br>Oopherectomy for ovarian cyst<br>Polyps resected and uterus biopsy<br>Transvaginal ultrasound for ovarian cyst (n=2)<br>Upper and lower GI endoscopy for stomach mass and sigmoid diverticulosis

MRI = magnetic resonance imaging, LV = left ventricular, EUS FNA = endoscopic ultrasound fine needle aspiration, FNA = fine needle aspiration, GI = gastrointestinal

[1]Relevant questionnaire data or additional correspondence on invasive procedures were available from 18 participants with serious final diagnoses, and 144 participants with non-serious or uncertain final diagnoses
