# Supplementary material for: Impact of detecting potentially serious incidental findings during multi-modal imaging
Source: Wellcome Open Res. 2018 Aug 2;2:114. Originally published 2017 Nov 30. [Version 3] doi: 10.12688/wellcomeopenres.13181.3 (PMC6024231; doi:10.12688/wellcomeopenres.13181.3)
Supplement: Supplementary file 9 [file wellcomeopenres-2-16045-s0008.tgz › 1376bb44-e93f-41df-9c7a-b26ff543d19b.pdf]

Supplementary File 9: Clinical management (medication and procedures) of the 21 participants with potentially serious incidental findings which were finally diagnosed as serious

Modality	Final diagnosis (one participant per diagnosis unless otherwise indicated)	Clinical management
Brain MRI	Arachnoid cyst with hydrocephalus	Neurosurgical drainage
	Meningioma compressing brainstem	Excision
	Pituitary tumour (n=2)	Transsphenoidal resection (n=1)
		Referred to specialist, no data on interventions (n=1)
Cardiac MRI	Atrial fibrillation	Warfarin
	Hypertrophic obstructive cardiomyopathy	Referred to specialist, no data on interventions
	Cardiomyopathy	Coronary angiogram, ramipril
	Coronary heart disease	Stress echocardiogram, coronary angiogram, aspirin, beta-blockers
	Heart block and LV impairment	Beta-blockers
	Lung tumours (n=3)	Excision and chemotherapy (n=1)
		Biopsy (n=1)
		Excision (n=1)
	Thoracic aortic aneurysm (n=5)	Repair (n=2)
		Referred to specialist, no data on interventions (n=1)
		No data (n=2)
Body MRI: Abdomen	Abdominal aortic aneurysm	No data
	Gastrointestinal stromal tumour	Excision
	Pancreatic neuroendocrine tumour	Excision and enzyme supplements
DXA	Osteoporotic crush fracture	Prophylactic medication

– = Not reported, MRI = magnetic resonance imaging, LV = left ventricular, EUS FNA = endoscopic ultrasound fine needle aspiration, DXA = dual energy X-ray absorptiometry
