# Supplementary material for: Impact of detecting potentially serious incidental findings during multi-modal imaging
Source: Wellcome Open Res. 2018 Aug 2;2:114. Originally published 2017 Nov 30. [Version 3] doi: 10.12688/wellcomeopenres.13181.3 (PMC6024231; doi:10.12688/wellcomeopenres.13181.3)
Supplement: Supplementary file 10 [file wellcomeopenres-2-16045-s0009.tgz › 0d98bef7-82b3-4bbc-9722-f8ad992c5dc3.pdf]

**Supplementary File 10: Rates of radiographer flagging and rates of radiologist confirmation of potentially serious incidental findings in the first 7000 imaged UK Biobank participants**

| Participant blocks of 1000 (in order of attendance) | Flagged by radiographers[1] | | Confirmed by radiologists[2] | |
|---|---|---|---|---|
| | N participants | % of total imaged | N participants | % of total imaged |
| 1-1000 | 66 | 6.6 | 18 | 1.8 |
| 1001-2000 | 35 | 3.5 | 19 | 1.9 |
| 2001-3000 | 61 | 6.1 | 27 | 2.7 |
| 3001-4000 | 30 | 3.0 | 16 | 1.6 |
| 4001-5000 | 22 | 2.2 | 14 | 1.4 |
| 5001-6000 | 11 | 1.1 | 7 | 0.7 |
| 6001-7000 | 27 | 2.7 | 19 | 1.9 |
| Mean per 1000 participants | 36 | 3.6 | 17 | 1.7 |

[1] Chi-square 72.5, 6 degrees of freedom, p<0.0001

[2] Chi-square 13.0, 6 degrees of freedom, p=0.04
