# Supplementary material for: Impact of detecting potentially serious incidental findings during multi-modal imaging
Source: Wellcome Open Res. 2018 Aug 2;2:114. Originally published 2017 Nov 30. [Version 3] doi: 10.12688/wellcomeopenres.13181.3 (PMC6024231; doi:10.12688/wellcomeopenres.13181.3)
Supplement: Peer review history

# Peer review: Referee response for version 2

**DOI:** 10.21956/wellcomeopenres.14912.r33394

**Published:** 2018-06-27

**Authors:** Eline M Bunnik

**Affiliations:** 1 Department of Medical Ethics and Philosophy of Medicine, Erasmus MC, University Medical Centre Rotterdam, Rotterdam, Netherlands

This much-awaited paper reports the experiences of UK Biobank, one of the largest research imaging efforts, with incidental findings. The results are of relevance to other research imaging groups around the world, and makes a valuable empirical contribution to evolving ethics and policy discussions on the management of incidental findings in research imaging contexts. A strength of this paper is that it monitors both the clinical impact and the psychosocial impact of the feedback of incidental findings on research participants. Also, the results of this study have been used to improve the informed consent process of UK Biobank (p. 12). The paper is nicely and clearly structured and comprehensive. 

 I have three - not too major - concerns with this paper

 - The authors claim at several points in the text that e.g.  "limited data exist" on the clinical and other implications of learning about incidental findings on research participants and that "data (...) are scarce" but would be much welcomed to inform the debate on appropriate protocols for handling incidental findings (page 3). Thus, the authors seem to suggest that their study is "the first" (page 12) to have looked into these implications empirically. This is not the case. The authors may have overlooked some of the available evidence ^1^^-^^2^ and should either discuss this evidence or rephrase sections of the paper in which they suggest that there is little evidence.

 - At times, the ethical argumentation falters a little. For instance, in the introduction the authors state that in research studies, potential harms should be minimised. This is correct, but a reference might clarify the scope and nature of this assumed obligation, as there are many different conceptions and interpretations of this obligation. Also on page 11, references are missing when the authors are discussing the principles of beneficence and non-maleficence and respect for autonomy. On page 12, it is argued that (the many) false positives (associated with systematic radiologist review) will make research participants (and society) worse off through unnecessary follow-up testing, while false negatives do not make participants worse off. I do not agree. False negatives can lead to false reassurance, which may pose health risks. The authors say that the participant information materials now explain more clearly how participation in UK Biobank does not constitute a health check (page 12). However, I am concerned that a subgroup of participants will still believe or expect their images to be reviewed for abnormalities, and will thus run the risk of false reassurance. Also, there is a difficulty that the harms associated with false positives are felt on a societal level (the costs and the efforts involved in (often unnecessary) follow-up), but not on an individual level: 97,7% of participants "reported being glad to be told about their potentially serious" incidental findings (p. 10). Thus, the authors thus slightly downplay the harms associated with false negatives and highlight the harms associated with false positives. Their conclusion that radiographer flagging is better than systematic radiologist review (with a lower rate of false positives) does not come as a surprise, but may based on a - in my view - slightly skewed weighting of benefits and harms. However, I do agree with the authors that researchers' obligations are mostly to meet the requirements detailed in the informed consent process, and also that there are good pragmatic reasons for UK Biobank to opt for a radiographer flagging policy, and that this is acceptable as long as the consent process is careful and effective in conveying that images are not being checked for abnormalities. 

 - And a final question: on page 4, it is explained that "radiographers were trained in the relevant imaging protocols but did not receive specific training in image interpretation". In a paper that prof.dr. Meike Vernooij and I wrote some time ago ^3^, we argued that whether an incidental findings is detected (in this context: whether and what kinds of findings will be flagged) will depend upon various technical, social and organisational factors, including the training, message, or instructions given to the radiographers. For this reason, I am curious to know what was said/what is being said to the radiographers by the project leads (e.g. "If you see something, you should notify X. Do try not to see things. Remember, this is a research study, not a clinical setting. Check the images for quality only, try not to look at any potential abnormalities." or something very different). May be the authors can add one sentence to the section on the two protocols to explain e.g. whether or not radiographers were discouraged from noticing findings or any other relevant variables in the instructions given to radiographers. Providing these details to research participants as part of the consent process could also be a way of conveying to participants that the research imaging does not constitute a health check. 

 Overall, I support the indexing of this paper.

I have read this submission. I believe that I have an appropriate level of expertise to confirm that it is of an acceptable scientific standard.

## References

[1] BosDPoelsMMAdamsHHAkoudadSCremersLGZonneveldHIHoogendamYYVerhaarenBFVerlindenVJVerbruggenJGPeymaniAHofmanAKrestinGPVincentAJFeeldersRAKoudstaalPJvan der LugtAIkramMAVernooijMW: Prevalence, Clinical Management, and Natural Course of Incidental Findings on Brain MR Images: The Population-based Rotterdam Scan Study. *Radiology*.2016;281(2) : 10.1148/radiol.2016160218 507-515 10.1148/radiol.2016160218 27337027

[2] de BoerAWDrewesYMde MutsertRNumansMEden HeijerMDekkersOMde RoosALambHJBlomJWReisR: Incidental findings in research: A focus group study about the perspective of the research participant. *J Magn Reson Imaging*.2018;47(1) : 10.1002/jmri.25739 230-237 10.1002/jmri.25739 28470774

[3] BunnikEMVernooijMW: Incidental findings in population imaging revisited. *Eur J Epidemiol*.2016;31(1) : 10.1007/s10654-016-0123-0 1-4 10.1007/s10654-016-0123-0 26861155PMC4756040

# Response

**Published:** 2018-07-27

**Authors:** Lorna Gibson

**Affiliations:** University of Edinburgh, UK

We thank Dr Bunnik for taking the time to read our manuscript and for providing helpful comments on several aspects of our work.

 In particular, thank you for suggesting that we add a reference to the paper De Boer et al.[1] We became aware of this work after our manuscript was sent out for initial peer review; we appreciate the need to update our text, and we have added the reference accordingly. Similarly, we were aware of the work of Bos et al.[2] as we are conducting a systematic review of the prevalence of incidental findings on brain and body imaging.[3] We state in our introduction that **‘**limited data exist on the impact of feedback of IFs on participants[4] and health services[5]’[6] with references to studies of the psychological[4] and economic impacts,[5] and we agree with Dr Bunnik that a reference to Bos et al. would be suitable here, and have added this to the text. However, despite this additional reference, we do think there remains very limited robust empirical data on the impact of feedback of IFs; while we do not provide a comprehensive review of the published evidence here, we hope to describe this in forthcoming manuscripts.

 We appreciate that a large body of literature exists on the obligations of researchers to research participants, and on the ethical principles of beneficence, non-maleficence and autonomy. Following an initial peer review from Professor Bjorn Hoffman, we expanded on the particular ethical and legal background from which the UK Biobank IFs protocol was developed. We agree with Dr Bunnik that a lack of feedback of IFs may be misunderstood by some participants as false reassurance of health, and UK Biobank continue to evaluate participants’ understanding of consent. UK Biobank does not use questionnaires of participants and their general practitioners to follow-up participants without potentially serious IFs to determine whether or not these represent ‘false negatives.’ As such, we feel that we do not downplay the harms of false negatives, but simply do not have the data to comment on these at present. We also agree with Dr Bunnik that the economic impact of false positive IFs constitutes an important harm, and while we do not present data here, it is the subject of a forthcoming manuscript. We hope that the data we do present here will contribute meaningfully to the ongoing discussion of the ethics of feedback of IFs, but feel that a more extensive discussion is beyond the scope of this current work.

 We agree with Dr Bunnik that the training and instructions given to the radiographers would potentially impact on the prevalence of IFs.[7] UK Biobank trains radiographers to acquire research imaging data and perform quality checks of the images at the time of the scan. If the radiographers happen to notice something on the scan that they think could be potentially serious (either a finding listed in Supplementary File 3, or a finding that  meets the UK Biobank definition of potentially serious), then they are instructed to flag the images for review by a radiologist. The radiographers are not instructed to actively look for, or to avoid looking for IFs, rather, they are instructed that if they happen to notice a concerning finding, they should flag it for review.

 We thank Dr Bunnik again for her review, and for stimulating an interesting discussion of aspects of our work.

 1. de Boer, A.W., et al., *Incidental findings in research: a focus group study about the perspective of the research participant.* J Magn Reson Imaging, 2018. **47**(1): p. 230-237.

 2. Bos, D., et al., *Prevalence, clinical management, and natural course of incidental findings on brain MR images: the population-based Rotterdam Scan Study.* Radiology, 2016. **281**(2):507-515.

 3. Gibson, L., et al., *Potentially serious incidental findings on brain and body magnetic resonance imaging conducted among apparently healthy adults: a systematic review*, in *PROSPERO*. 2016. CRD42016029472.

 4. Schmidt, C.O., et al., *Psychosocial consequences and severity of disclosed incidental findings from whole-body MRI in a general population study.* Eur Radiol, 2013. **23**(5):1343-51.

 5. Sandeman, E.M., et al., *Incidental findings on brain MR imaging in older community-dwelling subjects are common but serious medical consequences are rare: a cohort study.* PLOS ONE, 2013. **8**(8):e71467.

 6. Gibson, L.M., et al., *Impact of detecting potentially serious incidental findings during multi-modal imaging [version 2; referees: 2 approved, 1 approved with reservations].* Wellcome Open Res, 2018. **2**:114.

 7. Bunnik, E.M. and M.W. Vernooij, *Incidental findings in population imaging revisited.* Eur J Epidemiol, 2016. **31**(1): p. 1-4.

# Peer review: Referee response for version 2

**DOI:** 10.21956/wellcomeopenres.14912.r33052

**Published:** 2018-05-08

**Authors:** Saurabh Jha

**Affiliations:** 1 Department of Radiology, Perelman School of Medicine, University of Pennsylvania, Philadelphia, PA, USA

I have read this submission. I believe that I have an appropriate level of expertise to confirm that it is of an acceptable scientific standard.

# Peer review: Referee response for version 1

**DOI:** 10.21956/wellcomeopenres.14300.r28579

**Published:** 2018-01-08

**Authors:** Saurabh Jha

**Affiliations:** 1 Department of Radiology, Perelman School of Medicine, University of Pennsylvania, Philadelphia, PA, USA

The paper is unique in that it quantifies the trade-offs between radiologists screening for incidental findings versus radiographers. The findings are not surprising -  radiologists detect more true positives but also more false positives. The scale of the difference is surprising. The analysis is granular and the discussion is robust. The authors have anticipated many criticisms, and preemptively addressed them.

 The paper would be strengthened by three additions:

  

1. A comparison of the operating characteristics of radiologists and radiographers graphically.
2. A tabulation of the serious incidental findings picked up by both groups. In particular, a clearer explanation of what the radiographers missed.
3. A brief explanation of how they concluded that letting radiographers screen leads to less net harms – I get it, intuitively, but many might be tempted to argue, and since this is a key point, how the authors arrived at this conclusion should be better explained. An economic model isn’t needed, but expansion of some examples would help.

I have read this submission. I believe that I have an appropriate level of expertise to confirm that it is of an acceptable scientific standard.

# Response

**Published:** 2018-04-26

**Authors:** Lorna Gibson

**Affiliations:** University of Edinburgh, UK

We would like to thank Dr Jha for his comments.

 Dr Jha suggested that we provide a comparison of the operating characteristics of radiologists and radiographers as a figure and we wondered if perhaps Dr Jha would like us to provide a figure showing a receiver operator characteristics curve? If so, we have deliberately chosen not to display such a figure, as it may give the misleading impression that systematic radiologist review of research images is a ‘gold standard’ protocol to which radiographers are being compared. Our article does not attempt to define one protocol as the ‘gold standard’ or ‘best’ protocol. Instead, we feel that there is no single ‘best’ protocol for handling PSIFs, rather, there will be more, and less, appropriate protocols depending on the imaging context. Our article therefore focuses on describing and weighing up the impacts, benefits and harms of each protocol in order to determine which is most appropriate to apply within the specific research context of the UK Biobank imaging study of 100,000 largely asymptomatic participants. We apologise if we have misunderstood Dr Jha’s comment, and we would be more than happy to readdress this point if so.

 The serious final diagnoses detected under each protocol are tabulated in Supplementary File 7. In brief, systematic radiologist review resulted in 21 serious final diagnoses. Radiographer flagging detected five of these 21 serious final diagnoses (one arachnoid cyst with hydrocephalus, one meningioma compressing brainstem, and three thoracic aortic aneurysms), and missed 16/21 (two pituitary tumours, two thoracic aortic aneurysms, three lung tumours, two cardiomyopathies, and one each of: atrial fibrillation, coronary heart disease, heart block with left ventricular impairment, abdominal aortic aneurysm, gastrointestinal stromal tumour, pancreatic neuroendocrine tumour, and an osteoporotic crush fracture). We have added this text to our results section.

 Dr Jha asked for further explanation about how we concluded that radiographer flagging resulted in less net harm compared to systematic radiologist review of all images. We elaborate on this in our response to a related comment made by Professor Hofmann, and we hope that our approach addresses Dr Jha’s comments.

# Peer review: Referee response for version 1

**DOI:** 10.21956/wellcomeopenres.14300.r28578

**Published:** 2017-12-27

**Authors:** Bjorn Hofmann

**Affiliations:** 1 Norwegian University of Science and Technology (NTNU), Gjøvik, Norway; 2 University of Oslo, Oslo, Norway

Impact of detecting potentially serious incidental findings during multi-modal imaging WellcomeOpenResearch.

 This study investigates radiographer ‘flagging’ with radiologist confirmation of potentially serious incidental findings (IFs) compared with systematic radiologist review of images of brain, cardiac and body magnetic resonance, and dual-energy x-ray absorptiometry scans from the first 1000 imaged UK Biobank participants. The study assessed the number and types of potentially serious IFs detected and their final clinical diagnoses. The study also includes a qualitative assessment of participants experience and understanding of participation and findings.

 The study finds that radiographer flagging missed some serious diagnoses, but avoided adverse impacts for many participants with non-serious diagnoses, compared to systematic radiologist review. This makes the authors conclude that UK Biobank’s responsibility to avoid both unnecessary harm to larger numbers of participants and burdening of publicly-funded health services suggests that radiographer flagging is a justifiable approach in the UK Biobank imaging study.

 The study appears well conducted and is well reported. Figures and tables are informative and the manuscript is well structured. The findings are interesting and make new contributions to the field. This is a valuable study – also beyond the UK Biobank imaging study. In particular, data on final clinical diagnosis and the impact of feedback of IFs are scarce. The study is distinctive in assessing the number and types of potentially serious IFs detected and their final clinical diagnoses.  It is also quite unique in investigating the impact of providing feedback about potentially serious IFs on participants, their friends, families and health services, with respect to factors such as: clinical assessments undertaken; emotional wellbeing, finances, work and daily activities; and participants’ and their general practitioners’ opinions about receiving feedback.

 I have some detailed remarks, which hopefully can be helpful to the authors in improving the manuscript even further.

 The study used a list of potentially serious Ifs (presented in a supplementary file), however, they do not discuss the inclusion criteria for this list. For instance, which criteria exist for severity, accuracy, and actionability for the various conditions? How does this relate to feedback of Ifs in other fields, e.g., ACMG’s recommendations from 2013?

 The reader may also want a discussion on why radiographers “did not receive specific training in image interpretation,” and whether such training would alter the outcomes. Some indications are given (from the group’s experience beyond the first 1000), but competency gained from formal directed training may be different from practical experience (based on volume).

 From the text one may infer that radiologists in both groups had access to data collected during the imaging visit (on the participant’s age, sex, body mass index, self-reported smoking status, alcohol consumption, medical history and medications), but this is not completely clear. This can easily be made explicit.

 The authors classified the final clinical diagnoses as serious if the findings were likely to significantly threaten lifespan or have a major impact on quality of life or major body functions of the research participants. It is unclear how “significantly threaten” is interpreted. Is it a risk score? How does it balance the severity of the event and its probability?

 The authors’ claim that it is “often assumed that early observation on imaging of presumed disease (prior to clinical presentation) is inevitably beneficial” has recently been confirmed in a systematic review of the literature ^1^.

 It is not quite clear what is meant by: “We reconciled multiple responses on similar items from the three questionnaires by prioritising ‘yes’ responses and included data from coding of free text responses.” Careful reading explains this, but the authors may want to help the reader here.

 The ethical assessment is limited to the principle of non-maleficence. One could argue that this is a surprisingly narrow ethical analysis. Other issues, such as in professional ethics (with basis in deontological or virtue ethics), could easily be argued to be relevant as well. Moreover, the authors briefly mention the alternative of not returning information on IFs as part of this type of research (the UK Biobank imaging study), but refer to “legal advice” and “duty of care” to conclude that return of IFs are warranted. Given their findings (e.g., on lack of benefit and altruistic motivations), this conclusion may not be as obvious as the authors think and may need more elaboration to convince readers who are not part of or familiar to the project. Moreover, it also justifies some reflection on the relationship between legal and moral considerations. It is clear that this is not an article on the ethics of IFs, but when addressing ethical issues, which I strongly endorse, this should be done somewhat more elaborate.

 In general this is a well-planned and well-conducted study with interesting results that will make a nice reference in the field.

I have read this submission. I believe that I have an appropriate level of expertise to confirm that it is of an acceptable scientific standard, however I have significant reservations, as outlined above.

## References

[1] HofmannBSkolbekkenJ: Surge in publications on early detection. *BMJ*.2017; 10.1136/bmj.j2102 10.1136/bmj.j2102 28483750

# Response

**Published:** 2018-04-26

**Authors:** Lorna Gibson

**Affiliations:** University of Edinburgh, UK

We would like to thank Professor Hofmann for his comments.

 With regards to the UK Biobank lists of incidental findings (IFs) provided in Supplementary File 3, ^1^ Professor Hofmann asked us to clarify criteria used to select IFs for this list, such as severity, accuracy and actionability, and how the list relates to feedback of IFs in other fields, such as the American College of Medical Genetics (ACMG) recommendations from 2013.  

 The lists of IFs deemed potentially serious (i.e. for feedback), and those deemed non-serious were developed after discussion with radiologists, other relevant imaging reporting specialists, radiographers, members of UK Biobank’s Imaging Working Group, and with reference to work conducted by the German National Cohort (GNC) study. The GNC lists were developed specifically for the GNC imaging study, after review of the literature and discussion of best practice by radiologists familiar with the GNC research imaging sequences, and GNC ethical framework which aimed to feedback relevant findings, and not feedback irrelevant findings. ^2^

 At the time of the development of the lists of IFs, there were limited empirical data available on the prevalence and types of IFs that could be expected on the types of imaging to be conducted by UK Biobank. Furthermore, the available studies differed in their definitions of IFs, some, but not all, of which included concepts such as severity and actionability within their definitions. Therefore, to further inform on the prevalence and types of IFs which may be expected on imaging conducted by UK Biobank, we conducted a systematic review of potentially serious incidental findings (PSIFs, as per the UK Biobank definition) on brain and body magnetic resonance imaging. We will report this work within a separate manuscript.

 An ACMG working group generated a list of genetic mutations and recommended that these are sought out and reported when a laboratory performs any clinical exome or genome sequencing. ^3^ In contrast, the UK Biobank lists of IFs are certainly not used as checklists to purposefully seek out, or exclude, specific types of IFs by either the radiographers or radiologists. Rather, when a radiographer happens to see something abnormal on a scan, during image acquisition or quality assurance checks, or when a radiologist is reviewing a flagged image, they can refer to the lists in conjunction with UK Biobank’s definition of a potentially serious IF when judging whether any observed IF was potentially serious (i.e. for feedback to participants and their general practitioners [GPs]) or not.

 To address Professor Hofmann’s comment that, ‘from the text one may infer that radiologists in both groups had access to data collected during the imaging visit (on the participant’s age, sex, body mass index, self-reported smoking status, alcohol consumption, medical history and medications), but this is not completely clear,’ we have amended the relevant text to improve clarity.

 We classified final clinical diagnoses as serious if the findings were likely to significantly threaten lifespan or have a major impact on quality of life or major body functions of the research participants. Professor Hofmann asked how we interpret the term “significantly threaten.” There is a paucity of empirical evidence on the natural history and final diagnoses of IFs, ^4^ and to our knowledge there are no validated risk scores for quantitatively determining the risk to lifespan of particular IFs which are detected on research imaging. Our classification of final diagnoses as ‘serious’ is, as we mention in the limitations subsection of the discussion, a matter of clinical judgement. We also write that, as such, “‘serious’ final diagnoses may not actually shorten life span, or substantially impact on major body functions or quality of life in the 21 participants concerned, who were apparently healthy at the time of their imaging visits.” ^1^ Given this inherent subjectivity in the classification of serious final diagnoses, we measured the repeatability of the clinical judgements of final diagnoses severity, and demonstrated a very good level of agreement. Independently, a consultant physician and an experienced specialty clinical radiology trainee classified final diagnoses, and we report in our results section that these two doctors agreed in 172/179 (96.1%) cases, with the remaining seven cases easily resolved by discussion. ^1^

 We state that “it is often assumed that early observation on imaging of presumed disease (prior to clinical presentation) is inevitably beneficial, but data on final clinical diagnosis and the impact of feedback of IFs are scarce.” Professor Hofmann kindly directed us toward articles describing a surge in publications on early detection of disease, ^5^ and a systematic review which demonstrates that some common screening tests are not associated with a reduction in either disease-specific or all-cause mortality. ^6^ However, we have chosen not to add these references to the article for three reasons. Firstly, we wish to separate PSIFs (and IFs more generally) from the concept of early detection of disease, as our data demonstrate that the vast majority of PSIFs will not be finally diagnosed as a serious conditions, i.e. the majority do not represent early detection of disease.  Secondly, we wish to keep separate the concepts of screening programs from protocols for handling IFs detected during research imaging; whilst data on the benefits and harms of screening programs may be generalizable to the context of PSIFs, screening purposefully for a particular disease using a validated test is a different context to the non-optimized demonstration of an abnormality (which may or may not represent disease) on research imaging, although we accept that the populations undergoing screening and population-based imaging research (i.e. asymptomatic people) are similar. Finally, whilst a discussion of our results in the context of screening, early detection of disease, and overdiagnosis is of great interest to us, as researchers and clinicians, we wish to keep this article focused in its scope. 

 With regards to our methods section, Professor Hofmann commented that, ’it is not quite clear what is meant by: “we reconciled multiple responses on similar items from the three questionnaires by prioritising ‘yes’ responses and included data from coding of free text responses.” We would like to clarify this with examples. The two questionnaires sent to participants, and the questionnaire sent to their general practitioners (available online at: http://www.ukbiobank.ac.uk/resources/) all asked whether or not the participants had been referred to a specialist. The participant questionnaires have tick-box response options of ‘yes,’ ‘no’ and ‘don’t know.’ The GP questionnaire is different, and asks the GP to tick a box if that action has been taken (i.e. no tick may represent ‘no’ or ‘don’t know’). In addition, there are multiple free text fields available on all three questionnaires. Therefore, multiple responses may be available about specialist referrals, depending on the return of the questionnaires, and the completion of tick boxes and free text spaces. We therefore had to reconcile these multiple responses, and decided to prioritise ‘yes responses,’ in order to generate a maximum count. For example, if a participant responded that they did not know if they had been referred, but their GP ticked that they had been referred, we prioritized the ‘yes’ response of the GP, and coded the participant as being referred to a specialist. Similarly, if a participant indicated on their six-month questionnaire that ‘no,’ they had not been referred to a specialist, but had previously indicated ‘yes,’ they had been referred on their six-week questionnaire (either by ticking the box, or mentioning a specialist appointment in free text, or both), we coded the participant has having been referred to a specialist. This methodology maximizes the counts of types of follow-up and impacts and makes use of the maximum amount of data available. We have added a description of this methodology to the end of the document containing the questionnaires, hosted at http://www.ukbiobank.ac.uk/resources/, and we have added this link to the appropriate text in the methods section.

 Professor Hofmann asked why radiographers did not receive specific training in image interpretation. The UK Biobank is a research resource, and as such, is not aiming to provide any form of individual health service, including image interpretation. Accurate image interpretation, even by radiologists, is difficult in any case within the context of UK Biobank, given the lack of clinical information on current symptoms or signs, and the non-diagnostic nature of the research imaging. This is clearly evident from our results: the vast majority of PSIFs detected by radiologists are finally diagnosed as non-serious disease. ^1^ Within their typical roles in health services, radiographers are not trained to provide interpretation of cross-sectional imaging. Given the limitations of the research imaging, the difficulties in interpreting it (even by radiologists) and the typical role of the radiographers, rather than training radiographers to interpret multiple modalities of non-diagnostic cross-sectional imaging without any clinical information, UK Biobank opted instead to manage participants expectations of what could reasonably be expected. To this end, our consent materials state that the imaging is not a ‘health check,’ and lack of feedback does not constitute an ‘all clear,’ and we continue to evaluate participants’ understanding of consent with regards to feedback of PSIFs.

 Professor Hofmann stated that the ethical issues described in our article require some elaboration, including 1) some reflection on the relationship between legal and ethical  considerations, 2) further explanation of our how we concluded that the return of IFs are warranted after considering “legal advice” and “duty of care,” and 3) that principles other than non-maleficence, such as professional ethics, are relevant to our conclusions that radiographer flagging is the more appropriate IFs protocol in the context of the UK Biobank. Similarly, Dr Jha asked for further explanation about how we concluded that radiographer flagging resulted in less net harm compared to systematic radiologist review of all images. ^7^

 We thank Professor Hofmann and Dr Jha for these comments, and agree with Professor Hofmann’s further statement that while this article is not focused the ethics of IFs, these issues do need to be addressed and elaborated upon. UK Biobank have carefully considered the legal and ethical background with regards to feedback of PSIFs, and with input from its Imaging Working Group, its independent Ethics and Governance Council, representatives of its major funders (Wellcome Trust and the Medical Research Council), legal input from UK Biobank's legal counsel and from external legal counsel, and ethics advice from Professor Michael Parker of Ethox (who is also a co-author on this manuscript). Following the evaluation study, UK Biobank summarised the data on PSIFs and provided a detailed and lengthy interpretation of the results in the context of both the legal and ethical backgrounds in reports to their funders. Therefore, for readers’ convenience, we have summarized the key points of these reports by adding concise sentences to the discussion text to further describe the legal advice, duty of care, the relationship between the legal and ethical considerations, and justification for our conclusions. We hope that this approach addresses both Professor Hofmann’s and Dr Jha’s comments.

 1.         Gibson LM, Littlejohns TJ, Adamska L, et al. Impact of detecting potentially serious incidental findings during multi-modal imaging [version 1; referees: 1 approved, 1 approved with reservations]. *Wellcome Open Res. *2017;2:114.

 2.         Bertheau RC, von Stackelberg O, Weckbach S, Kauczor HU, Schlett CL. Management of incidental findings in the German National Cohort. In: Weckbach S, ed. *Radiological incidental findings*. Switzerland: Springer; 2017:57-70.

 3.         Green RC, Berg JS, Grody WW, et al. ACMG recommendations for reporting of incidental findings in clinical exome and genome sequencing. 2013; http://www.acmg.net/docs/ACMG_Releases_Highly-Anticipated_Recommendations_on_Incidental_Findings_in_Clinical_Exome_and_Genome_Sequencing.pdf. Accessed 15th January 2018.

 4.         Gibson LM, Sudlow CLM, Wardlaw JM. Incidental findings: current ethical debates and future challenges in advanced neuroimaging. In: Illes J, Hossain S, eds. *Neuroethics: anticipating the future*. Second ed. Oxford: Oxford University Press; 2017.

 5.         Hofmann B, Skolbekken JA. Surge in publications on early detection. *BMJ. *2017;357:j2102.

 6.         Saquib N, Saquib J, Ioannidis JP. Does screening for disease save lives in asymptomatic adults? Systematic review of meta-analyses and randomized trials. *Int J Epidemiol. *2015;44(1):264-277.

 7.         Jha S. Referee report for: impact of detecting potentially serious incidental findings during multi-modal imaging [version 1; referees: 1 approved, 1 approved with reservations]. *Wellcome Open Res. *2017;2:114.
